# Detection and Characterization of Invertebrate Iridoviruses Found in Reptiles and Prey Insects in Europe over the Past Two Decades

**DOI:** 10.3390/v11070600

**Published:** 2019-07-02

**Authors:** Tibor Papp, Rachel E. Marschang

**Affiliations:** 1 Institute for Veterinary Medical Research, Centre for Agricultural Research, Hungarian Academy of Sciences, Hungaria krt 21, H-1143 Budapest, Hungary; 2 Cell Culture Lab, Microbiology Department, Laboklin GmbH & Co. KG, 97688 Bad Kissingen, Germany

**Keywords:** lizard, bearded dragon, *Pogona vitticeps*, cricket, *Gryllus bimaculatus*

## Abstract

Invertebrate iridoviruses (IIVs), while mostly described in a wide range of invertebrate hosts, have also been repeatedly detected in diagnostic samples from poikilothermic vertebrates including reptiles and amphibians. Since iridoviruses from invertebrate and vertebrate hosts differ strongly from one another based not only on host range but also on molecular characteristics, a series of molecular studies and bioassays were performed to characterize and compare IIVs from various hosts and evaluate their ability to infect a vertebrate host. Eight IIV isolates from reptilian and orthopteran hosts collected over a period of six years were partially sequenced. Comparison of eight genome portions (total over 14 kbp) showed that these were all very similar to one another and to an earlier described cricket IIV isolate, thus they were given the collective name lizard–cricket IV (Liz–CrIV). One isolate from a chameleon was also subjected to Illumina sequencing and almost the entire genomic sequence was obtained. Comparison of this longer genome sequence showed several differences to the most closely related IIV, *Invertebrate* *iridovirus* *6* (IIV6), the type species of the genus *Iridovirus*, including several deletions and possible recombination sites, as well as insertions of genes of non-iridoviral origin. Three isolates from vertebrate and invertebrate hosts were also used for comparative studies on pathogenicity in crickets (*Gryllus* *bimaculatus*) at 20 and 30 °C. Finally, the chameleon isolate used for the genome sequencing studies was also used in a transmission study with bearded dragons. The transmission studies showed large variability in virus replication and pathogenicity of the three tested viruses in crickets at the two temperatures. In the infection study with bearded dragons, lizards inoculated with a Liz–CrIV did not become ill, but the virus was detected in numerous tissues by qPCR and was also isolated in cell culture from several tissues. Highest viral loads were measured in the gastro-intestinal organs and in the skin. These studies demonstrate that Liz–CrIV circulates in the pet trade in Europe. This virus is capable of infecting both invertebrates and poikilothermic vertebrates, although its involvement in disease in the latter has not been proven.

## 1. Introduction

The word “irido” is derived from Iris, the name of a Greek goddess who personified the rainbow. This is due to the “rainbow like” iridescence observed in heavily infected insects as mature virions accumulate within the cytoplasm of their infected cells in large paracrystalline arrays. In current taxonomy, the family *Iridoviridae* is divided into two subfamilies, *Alphairidovirinae* and *Betairidovirinae* [1]. The former contains three genera (*Ranavirus, Megalocytivirus* and *Lymphocystivirus*) whose members infect primarily ectothermic vertebrates, and the latter comprises three genera (*Iridovirus*, *Chloriridovirus* and *Decapodiridovirus* [2]) that infect mainly invertebrates such as insects and crustaceans. To avoid confusion, in this paper we will follow the suggestion of Vetten and Haenni [3], and generally members of the family *Iridoviridae* will be referred to as iridovirids (in short: IV) to distinguish them from the sensu stricto-invertebrate-iridoviruses (IIVs), which belong to *Betairidovirinae*.

Members of the family possess linear, double-stranded DNA genomes, which vary in size from approximately 100 kbp (genus *Ranavirus*) to over 200 kbp (genus *Iridovirus*). Iridovirid genomes are unique among animal viruses in that they are circularly permuted and terminally redundant. Many representatives have relevance for agriculture or nature conservation, but their inability to infect mammalian hosts somewhat limited the study of this diverse virus family, and especially that of the IIVs. Comprehensive reviews have been written about research progress on this group of viruses over the last decades [4,5,6].

The first invertebrate iridescent virus was reported in the mid 1950s from insects [7,8]. Further viruses were found in a wide range of invertebrates, mainly arthropods, but there have also been a few reports from other taxa (mollusks, an annelid and a nematode) [9,10]. For a while, the name of the host from which an IIV was first isolated was used in the nomenclature, later the viruses were assigned type numbers based on the chronological order of the reports [11]. The major capsid protein (*MCP*) gene used to be the part of the genome most commonly used for the classification of IIV isolates [12,13], and the largest number of IIV sequences in GenBank is from this gene. Many date back more than 20 years and were published in a single study [14]. In that study, a molecular comparison of fragments of the *MCP* gene of eighteen diverse isolates revealed that IIVs of the *Iridovirus* genus clustered into three groups/clades. The so called “crusteceoiridovirus group” contained two isolates, which were closely related phylogenetically, but had been isolated at distant locations from evolutionarily distant arthropod hosts, and later were grouped together in the species *Invertebrate iridescent virus 31*. The “oligoiridovirus group” contained a single established member, *Invertebrate iridescent virus 6* (IIV6; synonym: Chilo iridescent virus, CIV), which was initially isolated from a stem borer (*Chilo supressalis*) lepidopteran in Japan, but other isolates have also been reported. IIV6 was assigned as the type species of the genus. All other isolates, which were obtained from different insect hosts collected on five continents, clustered into the polyiridovirus group [10]. Subsequent studies added a few further sequences and branches to this tree [15]. The genus *Chloriridovirus* for a long time contained a single member, which was later assigned to the species *Invertebrate iridescent virus 3* (IIV3), and separated from the genus *Iridovirus* based on differences in phenotypic traits (e.g., larger particle size: 180 nm) and on its narrower host range [16]. However, the study of Wong et al. [17], based on the analysis of 26 core genes [18] demonstrated that IIV3 clusters with the polyiridovirus group, indicating that the genus *Chloriridovirus* may need to be re-evaluated, integrating the polyiridoviruses. This revision was further supported by MCP protein-based analyses of novel polyiridoviruses [19,20]. Most recently, viruses found in three distinct crustacean species have been proposed either to represent a novel genus (*Decapoiridovirus*) within the subfamily *Betairidovirinae* [21,22,23], or even cluster outside of it [24].

Natural transmission of IIVs has been recorded across insect orders and even phyla, and consequently several IIVs have been suggested as agents for pest control both as wild type viruses (e.g., [25,26,27,28]), or as a recombinant vector, encoding a toxin [29]. Patent infections of IIVs are often associated with macroscopic iridescence in the animals and are mostly lethal. However, non-lethal covert (or inapparent) infections are also common, and can manifest in reduced lifespans and/or reproduction rates [10]. Despite descriptions of IIVs in many different hosts on all continents except Antarctica, full genome sequences are currently only available from nine IIV isolates [6].

At the beginning of our sequencing studies, full genome sequences were available from only two viruses: IIV3 and IIV6 [16,27,30]. The genome of IIV6 was found to be ca. 212 kbp (unique portion) long with 28.6% G + C content and comprises 468 open reading frames (ORFs), of which 234 are non-overlapping. These ORFs were numbered from *001R* to *468R*, which indicates their orientation (left or right transcribing) in the genome as well. Core IIV genes were defined in IIV6 [31], which include those involved in 1) nucleic acid biosynthesis, e.g., *DNA polymerase* (*037L*), *RNA polymerase II* (*176R, 428L*), *RNAse III* (*142R*), a *helicase* (*161L*) and a *DNA topoisomerase II* (*045L*) gene; 2) nucleotide metabolism, such as *ribonucleotide reductase* (*085L, 376L*), *dUTPase* (*438L*), *thymidylate synthase* (*225R*), *thymidylate kinase* (*251L*) and *thymidine kinase* (*143R*) genes; 3) virion formation, such as the *major capsid protein* (*MCP* or *274L*) or the *myristilated membrane protein* gene (*118L, 458R*) and 4) other genes coding for proteins of known function including inhibition of apoptosis (e.g., *157L*, *193R*). Other notable non-core putative genes identified in IIV6 include an *NAD-dependent DNA ligase* gene (*205R*) and a putative homolog of the *sillucin* (*160L*) gene coding for a cysteine-rich antibiotic peptide, the first described viral antibiotic (VAB) [27,30].

Around the millennium, a new isolate was added to the oligoiridovirus group. This IIV was originally detected in insects bred for the pet trade in Europe and named cricket iridovirus (CrIV) or Gryllus bimaculatus iridovirus (GbIV) after its first known host [25,32], and its wide host range was demonstrated amongst different insect orders [25]. In addition to the primary findings in crickets, closely related viruses have been repeatedly detected in lizards [33,34]. It has been hypothesized that lizards become infected with the virus when fed IIV infected prey insects. In 2001, a German group reported the isolation of IIV-like viruses from the lung, liver, kidney and intestine of two bearded dragons (*Pogona vitticeps*) and a chameleon (*Trioceros* [*Chamaeleo*] *quadricornis*) and from the skin of a frilled lizard (*Chlamydosaurus kingii*) on viper heart cells (VH-2) at 28 °C [33]. The frilled lizard showed pox-like skin lesions and one of the bearded dragons had pneumonia. The other lizards had died with non-specific signs. Part of the *MCP* gene of the isolates was sequenced and had 97% identity to the nucleotide sequence of IIV6, and 100% identity to the nucleotide sequence of GbIV. A host-switch of this virus from prey insects to the predator lizards was postulated [33]. It was later demonstrated in our laboratory at the time that an IIV isolate from a lizard host is capable of infecting crickets [35]. Another study described the detection of similar viruses in skin swabs and organs from different amphibian species kept in captivity in several European countries [36]. Skin swabs as well as organ samples were found positive for IIV by PCR and/or virus isolation in 12 specimens originating from anurans representing five families (Ranidae, Hylidae, Dendrobatidae, Leptodactylidae and Bufonidae) as well as from a caudate (Lake Urmia newt; Salamandridae).

Detection of IIV in vertebrate hosts has been carried out by isolation in cell culture and by conventional PCR (nPCR) [33,35,36]. A qPCR, targeting a portion of the *MCP* gene was developed in our laboratory and was found to reliably detect and quantify IIVs of the oligoiridovirus group in tissues from vertebrate and invertebrate hosts both with high and low copy numbers [37].

The present paper summarizes parts of our work performed over the past two decades in connection with IIV isolates from reptiles and arthropods as well as diagnostic testing of reptiles, amphibians and arthropods. In addition to listing the species affected, it includes animal infection studies performed with invertebrate hosts (crickets) in order to compare the pathogenicities of several isolates, as well as transmission studies with vertebrate hosts (bearded dragons). New sequence data obtained from eight isolates from six vertebrate and two invertebrate hosts as well the draft genome sequence analysis of a chameleon isolate are also presented.

## 2. Materials and Methods

### 2.1. Iridovirus Isolates Used

Eight different IIV isolates, originating from four different lizard species, a scorpion and the prey cricket species fed to these pets were used for molecular biological comparison and for bioassays (Table 1). All of the viruses were isolated in reptile cell lines at 28 °C as described elsewhere [35].

### 2.2. Diagnostic Screening of Vertebrate and Invertebrate Samples for IIVs

Between 2009 and 2018, samples from a total of approximately 900 reptiles, amphibians and invertebrates submitted for diagnostic testing were regularly screened for the presence of IIVs. Between 2009 and 2013, testing was carried out by virus isolation and, in some cases, conventional PCR (nPCR) [35]. From 2014 onward, samples were screened by real-time PCR (qPCR) according to Papp et al. [37]. No background information was available for most of the samples submitted.

### 2.3. Cell Culture-Based Methods (Isolation, Propagation and Purification)

Isolation of viruses was carried out on iguana heart cells (IgH-2, ATCC:CCL-108) and/or Russell’s viper heart cells (VH-2, ATCC:CCL-140) depending on the origin of the sample. Cell types in closest phylogenetic relationship to the virus host were preferred. Small pieces of tissues or the cotton heads of swabs were sonicated in 3 mL Dulbecco’s modified Eagle’s medium (DMEM) (Biochrom AG, Berlin, Germany) supplemented with antibiotics. The samples were centrifuged at low speed (1500× *g*, 10 min) for the removal of cell debris and bacteria, then 200 µL of the homogenate was inoculated onto approximately 70% confluent cell monolayers in 30 mm diameter Cellstar^®^ tissue culture dishes (Greiner Bio-One GmbH, Frickenhausen, Germany). In cases in which centrifugation of the samples was insufficient to avoid contamination with bacteria, samples were filtered with 0.45 µm filters FP 30/0.45 CA-S (Schleicher and Schuell MicroScience, Dassel, Germany). After incubating for 2 h at 28 °C, 2 mL nutrient medium (DMEM supplemented with 2% fetal calf serum, FCS, 1% non-essential amino acids, NEA, and antibiotics, AB) was added to each dish. Cells were examined for cytopathic effects (CPE) approximately every third day with an inverted light microscope (Wilovet, Wetzlar, Germany), and dishes were frozen when extensive CPE was seen. Cultures showing no CPE were frozen after two weeks of incubation for blind passaging. Two additional passages were performed from each dish after a freeze and thaw cycle and low speed centrifugation.

For the purpose of the animal infection studies or the genome sequencing experiments, in which higher virus yield or pure concentrated DNA was required, IIV isolates were further propagated in 175 cm^2^ tissue culture flasks using the same cell line as described above. For the sequencing experiments, purification and concentration was performed by ultracentrifuge pelleting in a Beckman XL-90 instrument using a 60 Ti rotor. After a freeze and thaw cycle, cell debris was first removed from the cell culture supernatants by low speed centrifugation (1500 × g, 10 min, 4 °C), and viruses were then concentrated by ultracentrifugation (60,000× *g*, 2 h, 4 °C). Supernatants were decanted, and pellets were resuspended in PBS.

Infectivity titers of the isolates were determined on 96-well cell culture plates (Greiner Bio-One GmbH). Overnight cultures of the identical cell lines were infected at a confluence of approximately 70% with a 10-fold dilution row of the virus isolate in nutrient medium. Each well of the decanted plates was inoculated with 100 µL from each dilution step of the row, with four repetitions, as well as with virus-free control medium. Plates were checked for the appearance of virus specific CPE every third day and finally evaluated after two weeks. TCID_50_ titers were calculated using the Spearman-Kärber estimation formula [38].

### 2.4. Animal Infection Studies

#### 2.4.1. Cricket Bioassay

An iridovirus negative colony of field crickets (*Gryllus bimaculatus*) was established at the Institute for Environmental and Animal Hygiene at the University of Hohenheim in Stuttgart. The first animals were obtained courtesy of Dr. Regina Kleespies, Biologische Bundesanstalt für Land- und Forstwirtschaft, Darmstadt, Germany, who had already tested them negative for IIV. The crickets had no contact with other insects, were fed with salad and dog food (Matzinger Hundeflocken, Nestlé Purina Pet Care, Euskirchen, Germany) and were regularly tested negative for IIV by nested PCR.

**Figure 1:**
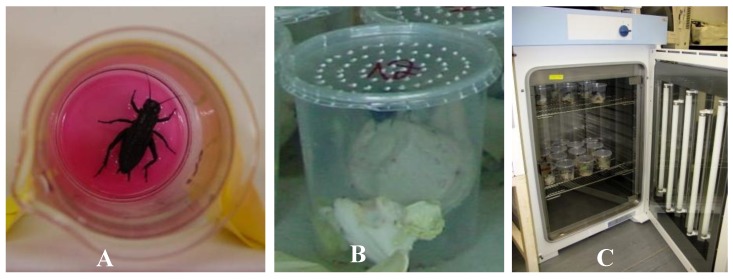
(**A**) Infection of a cricket by dipping into virus suspension. (**B**) Individual beaker with a cricket used in the infection studies. (**C**) Beakers were kept in a thermoregulated incubator with lights.

To study the virulence of IIVs from different hosts, a bearded dragon (*Pogona vitticeps*) “lizard” isolate (Ir.iso.3), a scorpion (*Pandinus imperator*) isolate (Ir.iso.8) and a cricket isolate (Ir.iso.7; Table 1) were propagated on IgH-2 to an equal titer of TCID_50_ 10^5.5–6^ /mL. Per os transmission experiments were carried out with a sub adult (last instar nymph) or young adult *Gryllus bimaculatus*. A total of 20 nymphs were completely immersed in virus suspension for approximately 30 s (Figure 1A). Ten crickets were dipped into IgH-2 supernatant as negative controls. In view of the cannibalistic behavior of this species, all animals were housed individually in beakers (diameter, 8.5 cm; height, 9.5 cm, Figure 1B). The animals were kept at either 20 °C or 30 °C in cooling–heating thermostat chambers with an adjusted 12/12 h light and dark cycle (Figure 1C). Tests were run for 60 days and mortality was recorded every 1–2 days, fresh food was provided as necessary (every 3–4 days). Fat body samples were collected from dead crickets and prepared for cell culture isolation, PCR and electron microscopy (EM). Half bodies of crickets were embedded in paraffin for histological examination. The surviving crickets at the end of each study were killed by snap freezing and decapitation and were also tested.

#### 2.4.2. Bearded Dragon Transmission Study

As parents for the bearded dragons used in the transmission study, six bearded dragons (three pairs) were bred. These animals were raised in separate facilities of the Institute for Environmental and Animal Hygiene at University of Hohenheim in Stuttgart from eggs. Their parents had tested negative for IIV in oral and cloacal swabs. All animals had also repeatedly tested negative for IIV in oral and cloacal swabs and had been previously fed IIV negative crickets only. Eggs were collected from these pairs and incubated at 28 °C in a Jaeger-Kunstglucke FB 50M incubator in the laboratory. The hatched bearded dragons had no contact to other reptiles and were fed IIV negative crickets from our own colony before the start of the study. Organs of five young animals that died soon after hatching were negative for IIV by nPCR and virus isolation.

Fifteen young bearded dragons at the age of five months were put into individual terrariums. The lizards were divided into three groups. The terrariums for negative control animals (NK) and infected animals (A and B) were kept in separate facilities away from each other and necessary hygienic measures implemented to avoid contamination of negative lizards during the infection trial. Animals were infected using the lizard IIV isolate Ir.iso.3 (Table 1) from a bearded dragon lung, propagated on IgH-2 cells to a titer of TCID_50_10^6.5^ /mL. Supernatant was purified first by centrifugation of the cell debris (see above) and further by three consecutive dialyzations against PBS for 20 h through 12 kDa cellulose membrane sacs (Spectrum Labs) in order to eliminate possible toxic or allergenic components of the supernatant. The virus titer in the purified suspension was checked again on IgH-2 cells and did not change with this treatment. For negative controls, DMEM supplemented with 2% FCS, 1% NEA and AB was treated the same way.

Two months before the beginning of the bearded dragon study, a batch of crickets was infected with the same lizard virus. Dead crickets were frozen and stored at −80 °C, the rest were killed at the end of the 60 day period and stored alike. In order to obtain a quasi-homogenous food-portion for each lizard, thawed crickets were dissected and predominantly, the iridescent ones were used in small cut pieces to feed the lizards. For the negative control lizards, negative crickets were prepared similarly.

**Figure 2:**
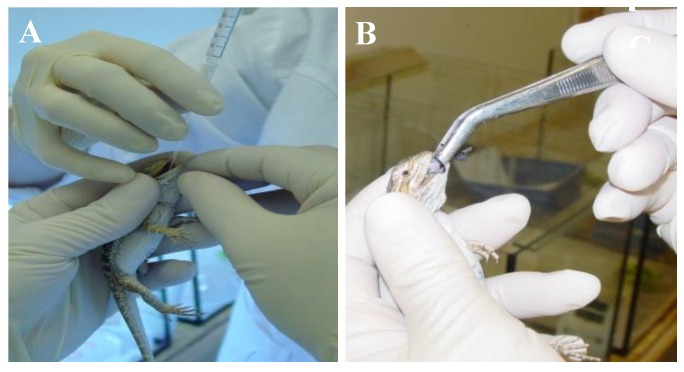
Transmission study of IIV in bearded dragons (*Pogona vitticeps*). (**A**) Administering virus preparate with an intra-gastric tube. (**B**) Force feeding with cricket-preparation. Note the bluish iridescence of the cricket preparate at the tip of the forceps.

Since the natural route of infection was unknown, several routes were chosen, including oral application of a cell culture isolate, feeding of infected crickets and intracoelomic injection. Bearded dragons of group A were given 1 mL of the virus containing solution administered through sterile cat catheters into the stomach (Figure 2A) and were force-fed with infected dead crickets (Figure 2B). Lizards in group B were also given virus suspension in the stomach and fed infected crickets, and an additional 1 mL of virus suspension was injected into their coelomic cavities. It was hypothesized that intracoelomic injection might provoke infection in the lizards if oral infection did not. Negative control lizards received the same treatment with the mock-solutions.

Animals were kept at 21–26 °C and 28%–38% relative humidity, using desert UVB fluorescent lamps without spot bulbs (in order to avoid an increase in body-temperature by basking which could adversely affect virus replication), and were checked daily. The lizards were fed with green salad and force-fed with the prepared crickets supplemented with mineral-vitamin powder (Korvimin ZVT + Reptil) every 2–3 days. Two weeks before the end of the study (from 46 days post infection (dpi)) no more crickets were fed. Oral and cloacal swabs were collected every week and changes in weight were also recorded (Supplementary Table S1). Swabs were examined for the presence of IIVs by cell-culture based and PCR methods. After 60 days, animals were euthanized by ketamine hydrochloride injection i.m. (Ursotamin, Bernburg AG, ca. 1000 mg/kg) and dissected. Gross pathology was performed and 12 different organs (brain, gonad, kidney, skin, lung, heart, fat-body, liver, tongue, stomach, ileum and colon) were collected for further assays as described above. Organs were collected under sterile conditions with different instruments used for the gastrointestinal tract than for all other tissues, and all instruments were washed in sterile distilled water, then in ethanol and flamed, then again in distilled water, before use on the next tissue in order to avoid contamination. The bearded dragon infection study received authorization from the Regierungspräsidium Stuttgart (No. V 195/2003) and was controlled by the Animal Welfare Committee of the University of Hohenheim.

#### 2.4.3. Microscopic Techniques

Following post mortem examination, organs from the bearded dragons as well as longitudinally dissected body halves of crickets in the transmission study were fixed for 48 h in Bouin’s solution of 714.3 mL/l picrin acid, 238.1 mL/l formalin (36%) and 47.6 mL/l acetate acid (all Merck, Darmstadt, Germany), and/or in 4% buffered paraformaldehyde solution and routinely processed for histology, cut at 3 µm and stained with hematoxylin-eosin (HE).

Examined organs of bearded dragons and fat body samples from selected crickets were fixed in 2.5% glutaraldehyde (Plano, Wetzlar, Germany) in cacodylate buffer (0.1 M sodium-cacodylate, pH = 7.4). After fixation, the samples were washed three times in sodium cacodylate buffer, and fixed in a 1% solution of osmium tetroxide in 0.1 M sodium cacodylate buffer for 1 h at 4 °C. The samples were washed three times with 0.1 M acetic acetate buffer, followed by contrast staining with a saturated uranyl acetate solution (5%) in 70% ethanol for 2 h at room temperature. Dehydration in an ascending row with alcohol followed. Propylene oxide (Serva, Heidelberg, Germany) was used as an intermediate matrix. The samples were then embedded in an epoxy resin combination (Plano, Wetzlar). After 48 h in an incubator at 60 °C, the blocks were polymerized, routinely cut, adsorbed onto 300 mesh copper grids and examined in a JEM-100SX electron microscope (Jeol LTD, Tokyo, Japan) at 80 kV by Prof. Dr. Alves de Matos in the Curry Cabral Hospital, Lisbon, Portugal.

### 2.5. Molecular Biological Techniques

#### 2.5.1. DNA Extraction

DNA was extracted from virus suspensions, sample homogenates of the animal infection studies, and from field samples submitted before 2013 with the DNeasy^®^ kit (Qiagen GmbH, Hilden, Germany) following the manufacturer’s protocols and using 100 µl of final elution volume. For diagnostic samples submitted after 2013, DNA was extracted directly from dry swabs or tissues using a commercial kit (MagNA Pure 96 DNA and viral NA small volume kit, Roche) according to the manufacturer’s instructions. DNA of the ultracentrifuge pelleted virions was extracted using the phenol-chloroform-isoamylalcohol method [39].

Concentration and purity of the DNA extracts (also of purified plasmids, see later) was determined with the spectrophotometric method (Ultrospec 2100 pro, Amersham Biosciences Europe GmBH, Freiburg, Germany and Nanodrop, ThermoFisher Scientific) at 230, 260 and 280 nm absorbance.

#### 2.5.2. Conventional PCRs (nPCR)

For comparative sequence studies, fourteen primers targeting six different genes were taken from the literature [40]. Further fifteen primers targeting *IIV6-VAB* (*160L*) and flanking genes as well as six primers targeting the *MCP* gene and flanking region were newly designed. Another thirteen primers targeting the DNA polymerase (*037L*) gene and twenty-three targeting the exonuclease (*012L*) gene were newly designed. All primer sequences are listed in a Supplementary File (Supplementary Table S2).

PCRs described previously [25,40] were performed according to the protocols described there. All other PCRs with novel primers were performed in 25 μL reaction mixtures, containing 1× concentration of Taq buffer with (NH_4_)_2_SO_4_, 1–3 mM of MgCl_2_, 200 µM of each dNTP, 1 µM from both forward and reverse primers and 0.5–1.25 U of Taq polymerase (all from MBI Fermentas, St Leon-Rot, Germany). The mixtures were amplified with an initial denaturation at 95 °C for 5 min followed by 35–45 cycles at 95 °C for 30 s, 45–60 °C for 30–60 s and 72 °C for 1−4 min. There was a final extension at 72 °C for 7 min. In the amplification cycles, the annealing temperatures for specific primers were set to T_m_ –4 °C, but not lower than the standard for the degenerated primers at 45 °C. Elongation times were set according to the expected product size, calculating 1 min for 1 kbp. Products were separated on 1%–1.5% agarose gels (Bioenzym, Oldendorf, Germany) in TAE puffer containing 0.5 µg/mL ethidium-bromide and visualized under 320 nm UV light.

#### 2.5.3. DNA sequencing (Sanger-, MPS)

Gel purified PCR amplicons (Invisorb Spin DNA Extraction Kit; Invitek GmbH, Berlin, Germany) and kit purified plasmids were sequenced using a BigDye Terminator Cycle Sequencing Kit v.3.1 or v.1.1 applying the PCR primers and pJET-F and -R primers for the PCR amplicons and cloned amplicons, respectively. Sequencing reactions were analyzed on an ABI prism 310 automated DNA sequencer (both Applied Biosystems, Foster City, CA, USA) at the Institute for Environmental and Animal Hygiene of Hohenheim University. Occasionally, commercial service providers (Biolux GmBH, Stuttgart; Eurofins, MWG Operon, Ebersberg, Germany; Biological Research Centre, Szeged, Hungary) were hired.

If direct sequencing of PCR products was unsuccessful or gave dubious results, then purified PCR amplicons were blunt-end cloned into pJET 1.2 vector (Fermentas, St Leon-Rot, Germany) following the manufacturer’s recommendations. Five μL of the ligated mixture was heat-shock transformed at 42 °C for 40 s into chemically competent *Escherichia coli* Top10F strain (Invitrogen, Life Technologies GmbH, Darmstadt, Germany). Transformed bacteria were plated on standard selective LB agar, containing ampicillin (100 µg/mL) and incubated at 37 °C. Selected colonies were propagated in selective LB broth and mini-preparations of the plasmid DNA were obtained with the alkaline method [41] or using a Plasmid Mini Kit (Qiagen GmbH, Hilden, Germany).

Multiple parallel sequencing (MPS) of the high casqued chameleon IV isolate (Ir.iso.1) was attempted from the ultracentrifuge pelleted virions. Phenol-chloroform extracted total DNA was sequenced by the Illumina technology by our service laboratory using the manufacturer’s recommendations (MiSeq platform at the Vet. Med Res. Inst, CAR, HAS, Budapest). CLC Genomics Workbench 8.5.1 was used for the downstream data analysis. After quality trimming of the paired-end reads, the corresponding algorithms of CLC Genomics were used to assemble de novo draft contigs, applying strict parameters (mismatch/insertion/deletion costs = 3 for each, length fraction = 0.8; similarity fraction = 1.0). Reads were also randomly mapped to the IIV6 reference sequence (NC_003038) using less strict criteria (no masking, mismatch cost = 2, insertion cost = 3, deletion cost = 3, length fraction = 0.5, similarity fraction = 0.8).

#### 2.5.4. Analysis of Sequences

Raw sequences obtained by Sanger sequencing method were processed by the ABI Sequence Analysis Program 5.1.1 (Applied Biosystems, Foster City, USA) then edited, assembled and compared using the STADEN Package version 2003.0 Pregap4 and Gap4 programs [42]. The sequences were compared to the data in GenBank (National Center for Biotechnology Information, Bethesda, USA) online (www.ncbi.nih.gov) using the BLASTN and BLASTX homology search programs. Homologous sequences were retrieved from GenBank, whereas the concatenated core genes alignment was retrieved from the current online International Committee on Taxonomy of Viruses (ICTV) report home page [1]. Multiple alignments of sequences were performed with ClustalW and MAFFT algorithms [43] of the Geneious and the CLC Main Workbench 8 programs using default settings. Phylogenetic molecular evolutionary analyses were conducted using MEGA version 10.0.2 [44], Topali v2.5 [45] and ExaBayes 1.5 [46] software, constructing Bayesian and maximum likelihood (ML) trees. Bayesian trees were constructed using the nucleotide (nt) and deduced amino acid (aa) alignments, selecting Hasegawa-Kishino-Yano (HKY) and Whelan and Goldman with gamma distribution (WAG + G) models, respectively [47,48]. Two independent runs were performed for 10^6^ generations. Every tenth tree was sampled out of which 25% was discarded as burn-in. SimPlot and BootScan analyses of the sequenced genes with their homologs retrieved from GenBank were performed using the SimPlot for Windows v.3.5.1 program.

#### 2.5.5. Real-Time PCR (qPCR)

Real-time PCR was carried out on diagnostic samples submitted after 2014 and on tissue and swab samples from animals in the transmission studies. Assays were carried out using primers, probe and conditions described previously [37]. Purified PCR products of the house cricket isolate (Ir.iso.7) and the clone of pJET plasmid inserted 1st round *MCP* products (primers F1 and R4) of a spiny tailed lizard IIV isolate (Ir.iso.4) were used as references in different dilutions for the quantification of viral DNA in the transmission studies [37].

## 3. Results

### 3.1. Diagnostic Testing

In addition to those in our previously published work [34,36,37], IIVs were detected in samples from 82 animals between 2009 and 2018 (Supplementary Table S3), including lizards, snakes, amphibians and insects. Of the samples tested by qPCR after 2014 (total 83), 32 (38.6%) were positive. Other viruses were also detected in several cases, most often adenoviruses (in 12 cases). In those cases in which a clinical history or pathology were provided, there were very variable changes noted, ranging from no clinical disease, to animals with general health issues (e.g., weight loss, apathy), to changes affecting their behavior, skin, intestines and/or liver, as well as central nervous signs. Sequences of nPCR products were identical to the corresponding sequences of CrIV (GbIV) partial *MCP* gene in all cases.

### 3.2. Sanger Sequencing Comparison of Isolates

**Figure 3:**
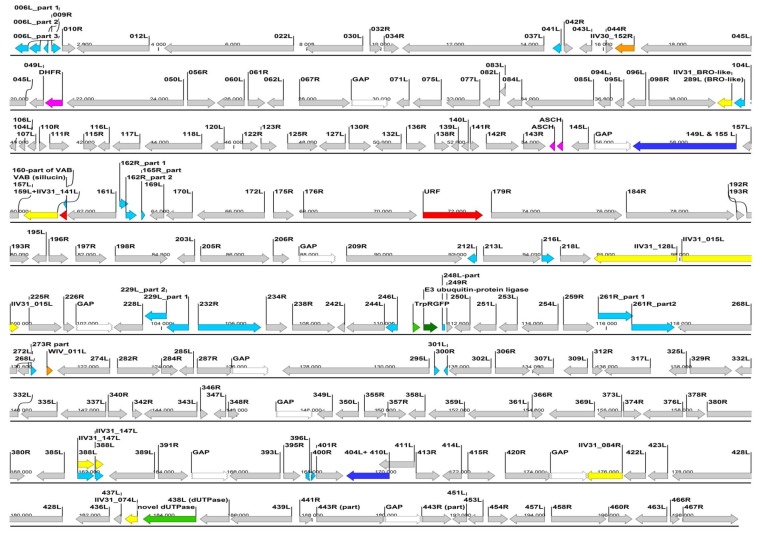
Putative genomic map of the chameleon IIV isolate (Ir. iso. 1) constructed based on nine contigs of an Illumina de novo assembly, and applying the ORF numbering of IIV6. Red dotted rectangles indicate the genome regions, which were earlier covered by a Sanger method based comparison of the isolates (please note that the gaps joining the contigs are not proportional to their actual probable size, but were arbitrarily set to 1 kbp). 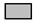 ORF highly similiar (85%–100%) to IIV6 homolog. 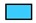 ORF broken or much shorter (>20%) than IIV6 homolog. 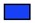 ORF is joined into one ORF from two IIV6 homologs. 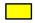 ORF most similar to an IIV31 (genus *Iridovirus)* gene/ORF. 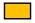 ORF most similar to a polyiridovirus (genus *Chlorididovirus*) gene. 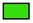 ORF most similar to a gene from a non-IV large DNA virus. 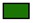 ORF most similar to a gene from a eukaryotic organism. 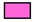 ORF most similar to a gene from a prokaryote. 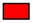 ORF with no homology to current GenBank entries. 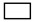 GAP (not yet sequenced part, arbitraty 1000, N’s joining of the mapped contigs).

We genetically characterized IIVs isolated over the course of six years from crickets and insectivorous pets fed with crickets (Table 1) based on complete and partial gene sequences. Full coding sequence (CDS) were obtained from eight genes: The *exonuclease II* gene (*IIV6-ORF012L* ortholog), the *DNA polymerase* gene (*037L*), *155L, 157L, 159L, 160L* (the *sillucin* viral antibiotic peptide gene, *VAB* gene), the major capsid protein gene (*MCP* or *274L*) and the upstream flanking *281R* (Figure 3). Partial CDS were determined from seven other genes: *ATPase* (*075L*), *helicase* (*161L*), *DNA-ligase* (*205R*), *thymidilate synthase* (*225R*), an apparently non-coding region (WIV *ORF011* homolog) downstream of the *MCP*, *ORF282R* and immediate early protein gene (*IE, ORF 393L*; see red dotted rectangles in Figure 3). Identity values in these regions, compared to corresponding IIV6 ORFs are shown in Supplementary Table S4. The overall summarized length of the determined portions for the different isolates exceeded 14 kbp.

The isolates included in this study were all identical to GenBank cricket iridoviruses: GbIV [32] and CrIV [40] based on the available partial *MCP* gene data. The studied isolates were found to be identical to one another by comparing sequences of several genes (*075L*, *160L* and flanking genes, *205L*, *225L*, *MCP* and flanking genes, *393L*). However, a very low inter-isolate variance (up to 0.4%) was detected in genes coding for DNA synthesis and degrading enzymes (DNA polymerase, exonuclease). The studied reptilian and invertebrate IIVs are therefore considered to represent a single virus type and are hereafter referred to as lizard and cricket IV variant (Liz–CrIV).

**Figure 4:**
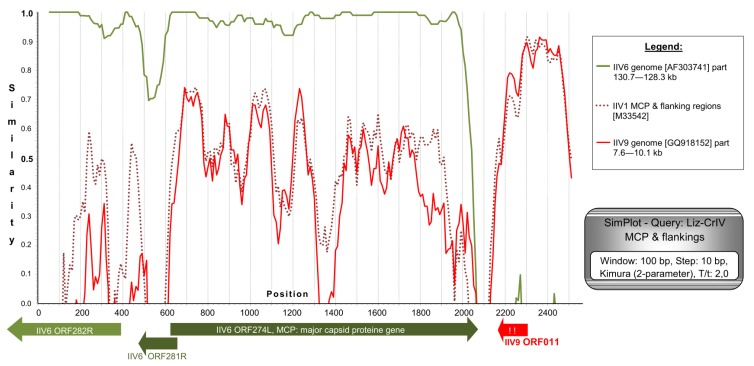
SimPlot analysis of the *MCP* gene with its flanking regions from our isolates. Three further members of the genus *Iridovirus* were available for the analysis in GenBank (IIV22 is highly similar to IIV1 and thus was omitted from the figure, for a better overview). Gene homologs with the highest BLAST values are drawn under the diagram. The region downstream of the *MCP* gene is a non-functional pseudogene homolog of IIV9 *ORF011*. Exclamation marks indicate stop codons in this pseudogene.

The *MCP* gene of Liz–CrIV with approximately 500 nt flanking regions on both sides (total of 2543 nt) was completely determined. CDS for the MCP was found to be 1428 nt (475 aa) long, and a putative recombination of ca. 500 nt was identified downstream of it. This correlates to the *MCP* flanking sequences of polyiridovirus group (genus *Chloriridovirus*) members (IIV1, -9, -22; Acc.Nos: M39542, GQ918152, M32799) and not those of IIV6 (Figure 4). A phylogenetic tree based on the complete *MCP* gene data has shown a considerably longer twig separating Liz–CrIVs from IIV6, than those projected from partial gene data (Supplementary Figure S1).

In the region of the *sillucin homolog* gene, the viral antibiotic peptide (VAB) found in IIV6, all of our eight isolates were found to be identical to one another. The putative protein product is 65 aa long (53 aa in IIV6). The N-terminal signal peptide sequence has three point mutations and a 7 nt long deletion near the cleavage site compared to *IIV6-VAB* (Supplementary Figure S2). This latter results in a frame shift and the putative translation of a longer (42/30 aa) non-homologous peptide, with no BLAST homology to any GenBank entry.

The adjacent ORF downstream showed even larger dissimilarity to its IIV6 homolog (*ORF159*). In the Liz–CrIV, this ORF is shorter (930 nt) than it is in IIV6 (1428 nt) and only its first third has high similarity to IIV6 *ORF159* (80%), whereas for its middle part counterparts were found in the IIV31 and IIV9 genomes. The second half of the gene has very limited (up to 30%) similarity to any known iridovirus sequence. This second and last third of *ORF159L* in the Liz–CrIV is a hypothetical recombination site (Figure S2). The following genes downstream are again highly similar to their corresponding ones in IIV6, but one is formed as a fusion of two IIV6 ORFs (*155L* and *149L*) due to a point mutation and loss of a stop codon.

### 3.3. Genome Sequencing of the Chameleon Isolate

The Illumina paired-end sequencing of the chameleon IIV isolate resulted in nine non-overlapping de novo assembled contigs (lengths between: 7.2 and 38.8 kbp), which contained altogether 190.5 kbp, presumably over 90%, of the complete Liz–CrIV genome. The coverage of these strictly assembled contigs was relatively low, around 100. Yet, the reliability of the assembly was substantiated by the fact that all previously acquired (Sanger methods) sequences (see above) were 100% identical to the matching regions assembled by the MPS method. An overall 89%–98% nt identity to the corresponding genome parts of IIV6 was found in the different contigs, with their GC content ranging from 26% to 29.5%. In each contig the order and orientation of the orthologous ORFs was apparently collinear with that of IIV6. Thus, a schematic map of the chameleon IIV genome was drawn by merging the contigs so that delineating unknown sequence “gaps” (runs of 1000 N’s) joined them (Figure 3). Based on the finding within each contig, we assumed a complete collinearity of the ortholog genes between the two viruses, and ORF numbers, where applicable, were kept according to the numbering of IIV6. The merged assembled genome sequence was submitted to GenBank and received the accession number: MN081869.

Regarding the core genes, in each case, BlastX gave the highest values to their IIV6 orthologs. Identity/similarity ranged from 88%/90% to 99%/100% in these protein sequences. Consequently, on the phylogenetic tree constructed based on the set of 25 core genes, Liz–CrIV and IIV6 clustered on two short twigs at the end of the same branch (Supplementary Figure S3). The apparent phylogenetic distance between them is larger than that of two variants (IIV22, IIV22A) and similar to that of two types (IIV22 and IIV30) in genus *Chloriridovirus*. Among ranaviruses, e.g., Grouper iridovirus (GIV) and Singapore grouper iridovirus (SGIV) show a comparable distance.

So far we have also identified 18 ORFs (*019R, 029R, 100L, 101L, 200R, 211L, 219L, 221L, 224L, 236L, 247L, 308L, 313L, 315L, 368R, 384L, 426R* and *468R*) that are missing in the Liz–CrIV, and 17 further ORFs (*006L, 009R, 041L, 162R, 165R, 212L, 216L, 229L, 232L, 246L. 261R, 273R, 300R, 301L, 322R, 388R, 396L* and *400R*) that are broken or truncated (Figure 3). In two cases (*149L* + *155L*, *404L* + *410L*) ORFs merged to single ones from two adjacent IIV6 orthologs. At least eight presumed recombination spots were detected in the genome, with the acquisition of 14 non-IIV6 orthologs, half of which have homologs in other IIVs, yet the rest are of supposed other viral, bacterial or eukaryotic origin (Table 2). Non-IIV homologs contain a second viral dUTPase with an intein splicing domain, a putative E3 ubiquitin protein ligase (similar to that in vertebrates), further algal and bacterial protein homologs and a 539 aa long putative protein with no BLAST homology to any current GenBank entry.

### 3.4. Cricket Infection Study

**Figure 5:**
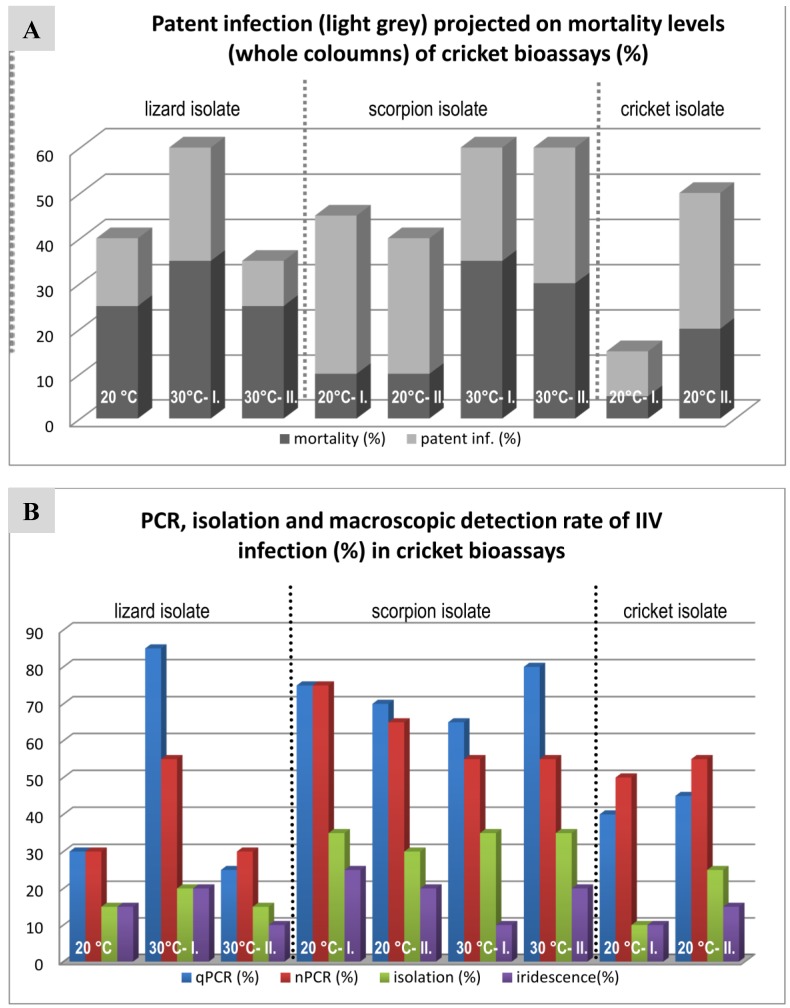
Graphic comparison of cricket bioassay results with different isolates at different temperatures. (**A**) Patent infection rates projected on mortality rates. (**B**) IIV infection rates detected by different methods.

The results of the cricket bioassay are summarized in Figure 5, Table 3 and are shown in detail in a Supplementary Figure S4. Unfortunately, due to technical problems with the cricket housing incubator, not all planned bioassays could be performed (e.g., cricket isolate 30 °C is missing). We found rates of mortality varying between 15% and 60% in the infected groups and 0% to 40% mortality in the negative groups. In the case of the negative control animals, however, the deaths of the crickets were never associated with virus propagation. All negative control animals were negative by cell-culture, nPCR and qPCR methods.

**Figure 6:**
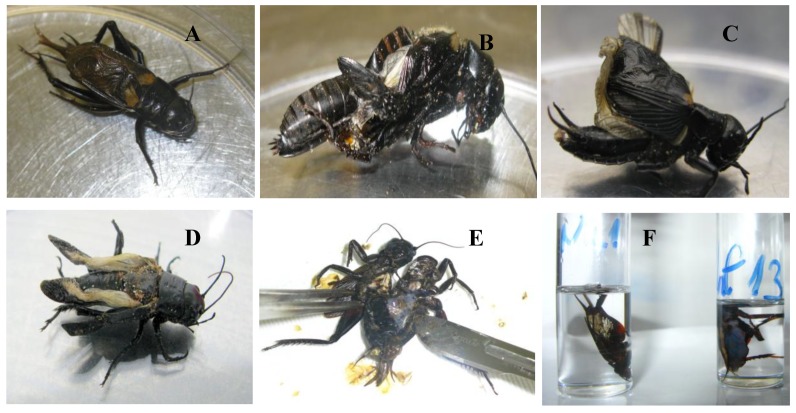
Malformations associated with IIV infection in crickets. (**A**) Negative control cricket. (**B**) Inability to complete ecdysis. (**C**,**D**) Distorted development of the wings in patently infected crickets. (**E,F**) Bluish iridescence in the fat body of patently infected crickets (on picture F right side of tube is infected, left is the negative control).

In the infected groups, apparent signs of infection were increased activity, swollen abdomen and molting abnormalities (Figure 6). The so-called “patently” infected crickets with very high virus loads and most often showing blue iridescence proved to have virus DNA copy numbers as high as 10^7^ to 10^10^ according to qPCR, while the so-called covertly infected animals had considerably lower (10^2^–10^4^) copy numbers in their fat bodies.

### 3.5. Transmission Study with Bearded Dragons

None of the bearded dragons included in the study showed any signs of clinical disease during the course of the study. They continued eating normally and gaining weight during the entire 60-day period (Table S1). There were, however, drops in body weight up to 12% recorded from one week to the other due to shedding. In group A, animal A5 started to lose weight (with 15% loss) during the second week and showed no appetite. It died emaciated on day 16 post infection (dpi) and was dissected. Gross pathology did not reveal remarkable alterations, except for a yellowish friable liver with glycogen deposits and lipidosis in histopathology.

Virus was detectable by isolation and PCRs from the oral and cloacal swabs of the infected animals from the first week after the beginning of infection, while the negative control lizards remained negative in these tests. The fourteen surviving animals were dissected at the end of the study. No changes were noted on gross pathology. A1 and A4 showed similar liver changes to those seen in A5.

No virus was detected in any of the negative control animals. In the infected animals, however, virus reisolation was successful mostly from the skin, the gastro-intestinal tract organs and in three cases in the liver and/or the brain as well. PCRs were positive from practically every organ (for details see Supplementary Figure S5). However, no specific macroscopic or histological changes were detected in any of these organs. EM was unable to detect virons in most tissues, but did detect individual virions in tissues with high viral copy numbers (according to qPCR) in individual cases. Comparing the virus loads (according to qPCR data) of these lizard tissues to those found in patently infected crickets showed three- to six-fold lower values, and the values of the gastro-intestinal tract organs and the skin were the highest in the infected bearded dragons (Figure S5).

## 4. Discussion

Our iridovirus studies were based on the findings of two German research groups. In 2001, isolation of IIV-like viruses was reported from the lung, liver, kidney and intestine of two bearded dragons (*Pogona vitticeps*) and a four horned chameleon (*Trioceros* [*Chamaeleo*]*quadricornis*) and from the skin of a frilled lizard (*Chlamydosaurus kingii*) [33]. Partial sequencing of the *MCP* gene of those isolates showed 100% identity to the nucleotide sequence of the cricket iridovirus CrIV [25,32]. In a parallel study by Marschang et al. [34], similar IIVs were isolated from two chameleons (*Trioceros, T*. [*Chamaeleo*] *hoehnelii*) and an iguana (*Iguana iguana*). The chameleons were cachectic, whereas the iguana had skin lesions. Later, our laboratory isolated further IIVs from insectivorous hosts (lizards and a scorpion) and from prey crickets (Table 1). A host-switch of this virus from prey insects to the predator lizards was postulated [33]. IIVs were also described in several different amphibian species [36]. In some cases, the animals were considered clinically healthy, while in others, increased mortality had been noted in a collection. Crickets fed to one of the amphibian groups also tested positive. Questions regarding the origin of this virus, its ability to switch hosts and infect vertebrates, its host range, possible transmission routes and genetic relationships among these viruses found in pet animals and other IIVs were raised by these previous reports, and our studies were undertaken to obtain answers to some of these questions.

An analysis of diagnostic testing for IIV in reptile, amphibian and insect samples in our lab from 2009 to 2018 shows that these viruses are still present in both captive bred insects and in reptiles and amphibians in captivity in Europe, and lengthens the list of species in which these viruses have been detected to include several new species including some snakes. It is, however, important to note that the results of some of this testing should be interpreted skeptically, since in many cases oral and/or cloacal swabs were tested. In cases in which vertebrates were fed infected insects, it is possible that virus detection in the oral cavity or even in the feces could reflect contamination from the insects rather than actual infection. On the other hand, tissues were also tested positive in several cases, including from animals that are not insectivorous, such as an Asian water monitor (Supplementary Table S3, No. 49) and an Indian rock python (Table S3, No. 57). It is not known how these animals might have come into contact with the virus. The majority of vertebrates in which these viruses have been reported are, however, wholly or occasionally insectivorous.

### 4.1. Comparison of Genome Fragments of Different IIV Isolates

The isolates included in this study (Table 1) were all identical to GenBank cricket iridoviruses: GbIV [32] and CrIV [40] based on the available partial *MCP* gene data. Additional genetic information was necessary in order to determine whether the viruses being diagnosed in vertebrate and invertebrate animals in the pet trade in Europe were related or represented a mix of viruses, possibly including invertebrate and vertebrate specific strains. The CrIV isolate kindly sent to our laboratory by Dr. Kleespies (Federal Biological Research Centre for Agriculture and Forestry, Institute for Biological Control, Darmstadt, Germany) has identical sequences to our isolates in the four analyzed genome regions (*012L, 037L, MCP, VAB*), and also in a PCR-restriction fragment length polymorphism (RFLP) analysis (author’s unpublished work). Despite the presence of several insertions, deletions and recombination sites throughout the genome of Liz–CrIV, all of our isolates studied were closely related or identical to one another in the studied genomic regions. Among our isolates there was a very low inter-isolate variance (up to 0.4%), only detected in two of the examined genes (*012L, 037L*). Since the isolates studied were obtained from six different species, including lizards and arthropods over the course of six years, this indicates that CrIV has evolutionarily stable variants circulating in Europe. In this paper we referred to it as lizard–cricket iridescent virus (Liz–CrIV) or simply CrIV, since its primary host is invertebrate and the infections of vertebrate hosts appeared due to (a) single or multiple host switch(es). Which insect species the original host could be is not clear in view of the finding that the recently disclosed genome sequence of a social insect [49], Jerdon’s jumping ant (*Harpegnathos saltator*), apparently harbors several mRNA and unmapped DNA sequences identical to our Liz–CrIV fragments. This suggests that this native Indian ant species is either a natural host for the CrIV or that it has been infected by the virus, e.g., during sample processing in laboratories in the USA or China [49,50]. This also shows that this virus is present on continents other than Europe.

The *MCP* gene sequence of Liz–CrIV is 1428 nt (coding 475 aa) long, the second longest reported in the family after IIV9 (484 aa; genus *Chloriridovirus*) [17] and shrimp hemocyte IV (SHIV, 477 aa; genus *Decapodiridovirus*) [21,23]. The MCP of Liz–CrIV is not only 8 aa longer than that of IIV6, but it has 13 additional mutation sites (Figure S1). This rate of MCP divergence (5.5%/4.5% for nt/aa) is smaller than that found between two separate types/species: E.g., IIV1 (Tipula iridescent virus; TIV) and IIV22 (Simulium iridescent virus; SIV; 19.8%/9.7%) [51], but much greater than those between two variants of the same type e.g., IIV22 and IIV22A (1.3%/0%) [52] or IIV31 and PjIV. Similarity plot analysis supported the hypothesis of recombination downstream of the *MCP* gene and the closest relationship of this region to the homologous regions of polyiridoviruses, e.g., IIV9 *ORF011*, adjacent to the IIV9 *MCP* gene (*ORF010*). In Liz–CrIV, however, this region is a pseudogene, a non-functional homolog of IIV9 *ORF011*, with two stop codons in the deduced protein sequence (Figure 4). The changes in the *VAB* sequence in Liz–CrIV result in a frame shift and a deduced active peptide sequence, which has no homology to any present GenBank entry. These changes indicate that the *VAB* gene in Liz–CrIV is functionally different from that found in IIV6. These changes in the gene, its transcriptional pattern, and its expression as a protein should be further investigated. The adjacent ORF downstream of *VAB* gene (*ORF159*), revealed another hypothetical recombination site (Supplementary Figure S2).

Despite the high similarity (97%) of the DNA polymerase gene of Liz–CrIV (*037L*) to its homolog in IIV6, the majority of the mutations and insertions in this gene clustered in close proximity, similar to the findings in the *MCP* gene. The structure and function of eukaryotic DNA polymerases have been characterized extensively [53], and were found to contain three active centers. The mutations of Liz–CrIV clustered in the region of the DEDDy domain, which is responsible for substrate specificity and DNA repair.

### 4.2. Genome Sequencing of the Chameleon Isolate, Differences from the IIV6 Genome

Based on the genetic differences between Liz–CrIV and IIV6 detected in the above described studies, it seemed worthwhile to attempt to determine the whole genome sequence of one of our IIV isolates. The chameleon isolate (Table 1, Ir.iso. 1) was chosen, as the one used in bioassays with both lizards and crickets. All core protein genes were covered by the assembly, thus a required state of the art phylogenetic tree reconstruction could be performed (Figure S3). As expected based on the previous studies, the Liz–CrIV clustered together with IIV6 in close proximity, yet the observed aa difference between the two viruses for this set of proteins was somewhat higher than 5% (5.4%). The overall nt identity between the determined part of the draft genome and the IIV homolog region was also found to be lower than the cut-off limit of 90% for the same virus species, at 76.9%. This is due to the numerous ORF deletions (18 missing and 17 broken or truncated) and ORF insertions (six from other IIVs, seven from other sources, Table 2), which will be discussed below.

The current taxonomical position of CrIV as a variant of IIV6 was based on biological and genetic data [40] with high similarity between the two viruses in susceptible insect host species and sequence data of seven homologous genes (93%–98% and 90%–97% identity for nt and aa). However, the genomic organizations of CrIV and IIV6 were earlier compared indirectly using RFLP [25] and were found to differ from one another. During our first studies comparing a more limited number of sequences from a higher number of isolates, lower (44%–98% and 46%–100%) sequence identities were found between our Liz–CrIV isolates and IIV6 across the examined genes. Early phylogenetic tree reconstruction based on *MCP* gene data (Supplementary Figure S1) has shown nt/aa differences smaller than those between two species (e.g., IIV1 and IIV22), yet larger than those between two variants (e.g., IIV22 or IIV31). In accordance with these findings the multiple parallel sequencing of the genome revealed that the set of core proteins differ from each other in the Liz–CrIV isolate and IIV6 a bit above the recently set [54] species demarcation criterion of 5%. Among the additional features to be considered for the demarcation, (1) phylogenetic relatedness, (2) a co-linear arrangement of genes, (3) similar genomic size and (4) similar G + C content [54] only genomic size difference (deletion of several ORFs) could be argued as a separating phenomenon between Liz–CrIV and IIV6. This is, however, not yet based on the full Liz–CrIV genome sequence, and final classification of Liz–CrIV as a species or an IIV6 variant should be postponed until the full genome sequence is available.

Virus evolution is achieved mainly by two processes: On one hand, random point mutations accumulate in the genome and eventually lead to a pool of virus mutants from which the most successful pathogen is selected by its ability to multiply best in a particular host organism. On the other hand, major alterations occur in the viral genome by recombination, deletion or insertion and can result in a virus with completely different properties in a very short period of time [55,56]. The limited number of mutational changes in the nucleotide sequences of the conserved ORFs and the evidence for multiple deletions and insertions/recombinations is compatible with the hypothesis that Liz–CrIV evolved relatively recently from IIV6.

What selection forces enabled the emergence of the Liz–CrIV is still a puzzle. Clues that could help solve it include the non-IIV6-like ORFs (Table 2), which occur in the Liz–CrIV genome and likely represent evidence of former recombination events. However, these insertions must be double-checked by PCR and Sanger sequencing in order to assure that they represent true genomic changes in the viral genome, and not assembly failures because of the relatively low coverage. Moreover, it is beyond the scope of the present paper to try and find answers for the important questions raised by these ORFs, e.g., (1) are these ORFs transcribed and translated in any or all Liz–CrIV infected cells? (2) What is their exact function and when do they switch on? This is relevant, since a number of these enzymes could play a role in the cell-cycle regulation (e.g., ASCH containing ORFs, IIV31_084R homolog) and/or the nucleotide metabolism (e.g., DHFR, IIV31_015R homolog, E3 ubiquitin protein ligase, dUTPase). (3) Why does the virus need a second *dUTPase* gene adjacent to its apparently functional IIV6 specific one (*ORF 438L*)? (4) Could the presence of an apparently reptile-related E3 ubiquitin protein ligase have any connection with the appearance of the Liz–CrIV in reptile hosts? Answering some of these questions will require experimental data, others can be answered using in silico analyses. There are reports in the literature proving that (regarding question 3), the same IIV genome can contain two active genes with the same enzymatic function [57], or (regarding question 4), that nucleocytoplasmatic large DNA viruses often contain host-derived genes [58,59].

### 4.3. Comparison of Three IIV Isolates in a Cricket Bioassay

The cricket bioassays were designed following previous studies in which extreme interassay variability was found in cricket mortality rates and in virus detection rates between trials with a single isolate (Table 1, Ir.iso.1; Table 3). We hypothesized that this variability could be due to changes in temperature between the individual trials. In addition, we hoped to determine biological differences between isolates from different hosts. However, standardization of the environmental temperatures (at 20 and 30 °C) did not lead to a clear reduction in the interassay variability (Table 3, for details see Figure 4), making the interpretation of differences between the isolates used for the cricket bioassays difficult to impossible. The mortality rates for all three isolates and both temperatures were all comparable to those from the previous study [35] and much lower than the 93% reported by Kleespies et al. [25] using the same infection method and a cricket isolate. However, the initial titer used by Kleespies was five-fold higher (2.2 × 10^11^ particles/mL) than that used in our studies, and was propagated in an insect cell line.

According to our data, the higher temperature (30 °C) does not seem to affect the ratio of patently or covertly infected crickets, although the mean survival time (MST) of the infected animals was slightly shorter at this temperature. The highest temperature at which IIV6 replication is still possible in in vitro systems is 32 °C [5], so it was of interest to determine whether a temperature close to that limit (30 °C) would adversely affect these lizard–cricket IIVs in their replication in vivo. The fact that it did not is also of interest considering the finding of these viruses in reptile species that thermoregulate to these and higher temperatures.

### 4.4. Bearded Dragon Transmission Study

IIV detection in lizards has been associated with a variety of clinical signs. These have included cachexia, keratoconjunctivits, hepatic hyperemia, splenomegaly, small white growths in the skeletal musculature, myxoid spindle cell sarcomas, liver lesions, apathy, CNS signs, bloody stool, hypoglycemia, sclerotic liver, stomatitis and sudden death [33,35,37]. Skin lesions have been reported in the greatest number of cases, including pox-like skin lesions, hyperkeratosis, loss of scales and cheilitis [33,37,60,61]. In some cases, virus has been detected in apparently healthy animals. Since IIVs have been detected in a variety of internal organs of lizards, it was hypothesized that these viruses infect the lizards. In many cases in which IIVs were detected, they were found in conjunction with other possible pathogens, including adenoviruses and ranaviruses [60,61]. The route of transmission for IIVs to vertebrates is unknown, but oral transmission via contaminated prey insects has been postulated [33]. Therefore, the bearded dragons were given both infected crickets and virus suspension per os. A second group also received virus suspension intracoelomically. Only one of the bearded dragons included in the study showed any signs of clinical disease during the course of the study. Virus was detectable by isolation and PCRs from the oral and cloacal swabs of the infected animals from the first week on after the beginning of infection. The finding of virus in oral and cloacal swabs was not surprising, since the alimentary route of infection was chosen, and cannot be interpreted as a sign of infection. However, continued detection after the animals were no longer being fed with virus positive crickets over a two week period indicates that virus either survived for a long period of time in the lizards or was replicating.

No direct connection was found between the early death of lizard “A5”, the smallest animal included in the study, and the virus inoculation. However, the successful re-isolation of virus from every tissue and the positive nPCR results suggested viremia. This was partly supported by the qPCR results. Solitary viral particles were found in several tissues in the EM examination. However, no viral assembly sites were detected, in contrast to the findings in infected crickets [35]. This indicates that virus replication in lizards is much lower than in invertebrates, a finding supported by the results of the DNA quantification by qPCR. The fact that CrIV [33,35], and even IIV6 [62] are capable of replication on poikilothermic vertebrate (reptile) cell lines below 30 °C supports this conclusion. It is also known that IIV6 inhibits cellular DNA, RNA and protein synthesis [63] and stimulates an immune response [64] in non-permissive vertebrate cells.

The highest viral loads were found in the gastrointestinal tract, with the highest overall concentrations found in the large intestine (colon) both by virus isolation and qPCR. This was expected due to the presence of possible remnants of the infected food-crickets, despite the two weeks of vegetable diet at the end of the study. The finding of relatively high virus loads by qPCR (and virus isolation) in the proximal portions of the digestive tract of some lizards, however indicates either a great capacity of these viruses to remain within the entire digestive tract over an extended period of time, or that virus replication took place here. Other organs (lung, heart, liver and skin) also had high virus loads (by qPCR) and in some cases, the virus was also isolated from these tissues. These findings indicate that virus did spread throughout the bodies of the infected lizards.

These results are in harmony with the findings in routine diagnostic lizard samples analyzed in recent years in our laboratory [37], including those presented in this paper (Supplementary Table S3) or other laboratories [33] where these organs were sources for IIV isolation from diseased lizards. Skin has also often been reported to be affected. However, no macroscopic or histological changes could be detected in any of these organs. The yellowish friable liver, as a sole pathological finding, resulted from glycogen deposition and lipidosis, and might be an indication of hepato-toxic effects of the viral proteins, as has been described in mice injected with IIV6 [65].

Comparing the virus loads (according to qPCR data) in the lizard tissues to those found in patently infected crickets infected with the same isolate, the values were in a three- to six-fold lower range, which could explain the difficulties encountered in finding the virus in the tissues in situ, since the sensitivity of EM is much lower than the sensitivity of e.g., virus isolation [35,66].

The results of these transmission studies support the hypothesis that CrIV-like viruses are able to infect vertebrates and replicate in a variety of tissues. However, when applied orally and intracoelomically, they did not induce disease. Since these viruses have been found together with other viruses in diseased lizards in several cases [60,61], including those described here, it is possible that they play a role in multi-factorial disease processes not mimicked in this study. The frequent detection of these viruses in skin samples and in animals with skin lesions could also indicate that virus transmission does not occur via the alimentary tract, but through wounds or imperfections in the skin. In invertebrates, IIVs often cause covert infections [5]. It is possible that this is also the case in vertebrates, and that patent infections only occur at low rates or under specific circumstances, e.g., low environmental temperatures. Further studies are necessary to better understand this.

## 5. Conclusions

A specific strain of IIV, here called Liz–CrIV has been shown to be circulating among invertebrates in the pet trade in Europe for at least the past two decades and is also regularly found in a wide range of poikilothermic vertebrate hosts including squamate reptiles and amphibians. This virus is closely related to IIV6, the type species of the genus *Iridovirus*, but shows several interesting differences in its genomic sequence, incorporating novel ORFs (potential genes) with diverse homology to other viral, prokaryotic and eukaryotic sequences, and mostly unknown functions, which remain to be analyzed in subsequent research projects. Transmission studies with crickets have indicated that several factors, including environmental temperature, influence the rate of virus replication and the development of disease in invertebrate hosts. Some of these factors have not yet been elucidated. Infection studies with bearded dragons indicate that viral DNA may replicate in vertebrate hosts, but could not be associated directly with disease, or that disease development may depend on unknown circumstances.

Evidence for the ability of these viruses to infect vertebrate hosts includes the regular detection of Liz–CrIV in diagnostic samples from vertebrates, detection of signification quantities of Liz–CrIV DNA as well as virus isolation from various tissues of bearded dragons following experimental infection, and the finding of a putative reptile-derived gene in the Liz–CrIV genome. Many details of the epidemiology of these IIVs in vertebrates as well as their possible impact on animal health require further study.

## Figures and Tables

**Table 1:** Invertebrate iridovirus (IIV) isolates used in sequencing studies and bioassays. Grey colored ID refers to laboratory notebook number, the final number is the year of collection. Of all positive organs listed, the origin of the isolates used in the sequence comparison is printed in italics. Isolates compared in a cricket bioassay are underlined.

	Host Species	ID, No.	Owner	IIV Positive Organs	Case History
Squamata,	High-casqued chameleon (*Trioceros* /*Chamaeleo/ hoehnelii*)	Ir.iso.1 100/2001	A	Kidney, liver, spleen, *lung,* intestine	Emaciation, kerato-conjunctivitis
	Bearded dragon (*Pogona vitticeps*)	Ir.iso.2 66/2003	B	*Lung,* heart, tongue	Unknown
	Bearded dragon (*Pogona vitticeps*)	Ir.iso.3 64/2003	B	*Lung*, brain, tongue, stomach, intestine	Unknown
	Spiny tailed lizard (*Uromastyx sp*.)	Ir.iso.4 08/2004	B	*Skin*	Hyperkeratosis
	Four-horned chameleon (*Trioceros quadricornis*)	Ir.iso.5 626/2000	C	*Liver*	Emaciation, several animals died suddenly
	Green iguana (*Iguana iguana*)	Ir.iso.6 1125/2000	D	*Skin*	Hyperkeratosis
Arthropoda	House cricket (*Acheta domesticus*)	Ir.iso.7 52/2003	E	*Whole body*	Prey insects, died suddenly
	Emperor scorpion (*Pandinus imperator*)	Ir.iso.8 **25/2006**	F	*Abdominal organs*	Loss of UV colouration

**Table 2:** Non-IIV6-like open reading frames (ORFs) in the chameleon IV sequence (names are according to those in Figure 3).

Name	Length in Liz–CrIV (aa)	Length of Homolog ORF (aa)	aa Identity/ Similarity (%)	Comment /Function
IIV30_152R	176	173	55/68	Hypothetical protein of IIV30
DHRF	158	159	50/73	Dihidrofolate reductase of *Labilithrix luteola* (Protobacterium)
IIV31_BRO-like	133	416	89/85	Ortholog of IIV31_198R and IIV6_189L C-terminal regions
ASCH containing /1	48	137	50/66	*Thermococcus sp*. ASCH and *Gonium pectorale* (alga) hypothetical protein
ASCH containing /2	53	137	53/69	ASCH containing protein (RNA binding) of Dependentiae bacterium
VAB (sillucin)	65	NA	NA	NO BLAST homology (only N-terminal part is similar to IIV6_160L (VAB = viral antibiotic peptide))
URF	539	NA	NA	NO BLAST homology to any GenBank entry (ca. 2kbp insert of foreign DNA, compared to IIV6 genome)
IIV31_128L	748	771	44/62	35%/55% identity/similarity to a hypothetical protein of Klosneuvirus
IIV31_015L	699	676	62/78	Polynucleotide kinase/ligase (PK) and a pseT superfamily domain
TrpRGFP	66	260	54/69	Tryptophan repeat gene family protein of an entomopoxvirus and a Kaumoebavirus
E3 ubiquitin protein ligase (RNFT1)	127	252	33/48	C-terminal half (63 aa) shows highest similarity to homolog gene of *Python bivittatus* and other snake homologs, it contains a ring-finger domain
IIV31_084R	318	269	57/74	Not completely covered, contains ABC type AA transport/ signal transduction system domain
IIV31_074L	115	116	37/52	Hypothetical protein of IIV31
Novel dUTPase	478	349	46/63	Most similar homolog found in Pithovirus; contains an intein splicing domain (N-terminal part), and a trimeric dUTPase domain (C-terminal part)

**Table 3:** Comparison of the (A) here described controlled-temperature cricket bioassay results with (B) those of three replicates (I–III) of an earlier study in our laboratory [35], which was performed with non-controlled temperatures.

**A.**									
	**Lizard Isolate**			**Scorpion Isolate**				**Cricket Isolate**	
	20 °C	30 °C I	30 °C II	20 °C I.	20 °C II.	30 °C I.	30 °C II.	20 °C I.	20 °C II.
**Mortality (%)**	40	60	35	45	40	60	60	15	50
**Patent inf. (%)**	15	25	10	35	30	25	30	10	30
MST* (days)	42	38.6	36.5	34	24.2	24.4	34.2	35	42
**qPCR (%)**	30 (15)	85 (15)	25 (5)	75 (10)	70 (5)	65 (15)	80 (20)	40 (40)	45 (20)
**nPCR (%)**	30 (15)	55 (15)	30	75 (5)	65 (5)	55 (25)	55 (25)	50 (10)	55 (10)
**Isolation (%)**	15	20 (5)	15	35	30	35	35	10 (5)	25
**Iridescence (%)**	15	20	10	25	20	10	20	10	15
**B.**									
	**Weinmann et al. [35]**								
	**I.**			**II.**				**III.**	
**Mortality (%)**	35			20				20	
**Patent inf. (%)**	NA			NA				NA	
**MST (days)**	NA			NA				NA	
**qPCR (%)**	NA			NA				NA	
**nPCR (%)**	75			15				30	
**Isolation (%)**	45			5				25	
**Iridescence (%)**	25			5				10	

*MST (mean survival time) was calculated for the proven patently infected animals. Data in brackets refer to samples with dubious results in the different tests: qPCR = 1–5 copies/µl detected, nPCR = faint bands on agarose gel, isolation = inconsistent results in the repeats. NA = not analysed.

## Supplementary Materials

The following are available online at https://www.mdpi.com/1999-4915/11/7/600/s1, Figure S1: Phylogenetic tree based on the complete *MCP* gene data, Figure S2: SimPlot analysis of the *sillucin* gene homolog with its flanking regions; Figure S3: Phylogenetic tree reconstruction of *Iridoviridae* based on 25 core genes; Figure S4: Detailed results of the cricket bioassay; Figure S5: Detailed results of the bearded dragon infection study; Table S1: Animals of the bearded dragon infection study; Table S2: Primers used for PCRs and Sanger sequencing; Table S3: IIV detection in diagnostic samples, 2009-2018, Table S4: Sequence identity values (%) between Liz-CrIV and IIV6.
