# Supplementary material for: Detection and Characterization of Invertebrate Iridoviruses Found in Reptiles and Prey Insects in Europe over the Past Two Decades
Source: Viruses. 2019 Jul 2;11(7):600. doi: 10.3390/v11070600 (PMC6669658; doi:10.3390/v11070600)
Supplement: Supplementary file 1 [file viruses-11-00600-s001.zip › IIV_Supplementary Table S3-diagnostic samples.docx]

**Supplentary Table S3 (continue next 5 pages)**: IIV detection in diagnostic samples, 2009-2018

| **Host order, family** | **No.** | **Year of detection** | **Lab No.** | **Species** | **Samples tested positive** | **Detection method^1^** | **Other viruses detected^2^** | **Case history** |
| --- | --- | --- | --- | --- | --- | --- | --- | --- |
| Squamata, Agamidae |  | 2009 | 71/09 | Bearded dragon (*Pogona vitticeps*) | Oral swab, cloacal swab | CC (IgH2) | AdV | Bloody faeces, enlarged and hardened liver |
|  |  | 2010 | 90/10 | Bearded dragon (*Pogona vitticeps*) | Skin | nPCR |  | Skin lesions (pustules) on the head |
|  |  | 2010 | 68/10 | Bearded dragon (*Pogona vitticeps*) | Swab | CC (IgH2) |  | Apathy, emaciation |
|  |  | 2010 | 97/4/10 | Bearded dragon (*Pogona vitticeps*) | Oral and cloacal swab | CC (IgH2) |  | Hepatopathy, ascites, enteritis |
|  |  | 2010 | 117/10 | Bearded dragon (*Pogona vitticeps*) | Swab | nPCR |  | Head tremor |
|  |  | 2010 | 158/3/10 | Bearded dragon (*Pogona vitticeps*) | Swab | nPCR | AdV | Clinically healthy, screening |
|  |  | 2010 | 158/4/10 | Bearded dragon (*Pogona vitticeps*) | Swab | nPCR |  | Clinically healthy, screening |
|  |  | 2010 | 158/5/10 | Bearded dragon (*Pogona vitticeps*) | Swab | nPCR |  | Clinically healthy, screening |
|  |  | 2010 | 158/6/10 | Bearded dragon (*Pogona vitticeps*) | Swab | nPCR |  | Clinically healthy, screening |
|  |  | 2011 | 7/11 | Bearded dragon (*Pogona vitticeps*) | Oral and cloacal swab | CC (IgH2) | AdV | Inappetence, apathy, ascites, monocytosis |
|  |  | 2011 | 64/1/11 | Bearded dragon (*Pogona vitticeps*) | Oral and cloacal swab | CC (IgH2) |  | Died suddenly, possible stomatitis |
|  |  | 2011 | 139/1/11 | Bearded dragon (*Pogona vitticeps*) | Oral swab, cloacal swab | CC (IgH2), nPCR |  | Cachexia, enlarged gall bladder |
|  |  | 2012 | 20/12 | Bearded dragon (*Pogona vitticeps*) | Skin crusts | nPCR | AdV | Crust around the mouth |
|  |  | 2012 | 91/12 | Bearded dragon 15(*Pogona vitticeps*) | Oral and cloacal swab | CC (IgH2, BC), nPCR | AdV | Presented for routine health check |
|  |  | 2012 | 136/12 | Bearded dragon (*Pogona vitticeps*) | Oral swab, cloacal swab | nPCR |  | Slime in oral cavity, mouth gaping, otherwise alert and responsive |
|  |  | 2013 | 22/13 | Bearded dragon (*Pogona vitticeps*) | Oral and cloacal swab | CC (IgH2), nPCR |  | Apathy and vomiting |
|  |  | 2013 | 41/13 | Bearded dragon (*Pogona vitticeps*) | Oral and cloacal swab | CC (IgH2) |  | CNS signs |
|  |  | 2013 | 50/13 | Bearded dragon (*Pogona vitticeps*) | Cloacal swab | CC (IgH2), nPCR | Ranavirus | Progressively worsening CNS signs (tremor, hypermetria, hyperaesthesia, delayed righting reflex) |
|  |  | 2013 | 68/13 | Bearded dragon (*Pogona vitticeps*) | Oral swab | nPCR |  | Dyspnea |
|  |  | 2010 | 168/10 | Common agama (*Agama agama*) | Liver and kidney | nPCR |  | Bad general condition, jaw abscess, osteolysis, lower jaw fracture, oxurids and choleoeimeria |
|  |  | 2012 | 41/12 | Agamid | Oral swab | CC (IgH2), nPCR |  | Distal limb paralysis |
|  |  | 2011 | 106/38/11 | Water dragon | Swab | CC (IgH2) |  | Not provided |
|  |  | 2015 | 1502S57253 | Bearded dragon (*Pogona vitticeps*) | Swab | qPCR |  | Not provided |
|  |  | 2015 | 1510S69617 | Bearded dragon (*Pogona vitticeps*) | Swab | qPCR |  | Not provided |
|  |  | 2017 | 1712S39161 | Bearded dragon (*Pogona vitticeps*) | Tissues | qPCR | AdV | Not provided |
|  |  | 2018 | 1807S34437 | Bearded dragon (*Pogona vitticeps*) | Tissues | qPCR |  | Not provided |
|  |  | 2018 | 1808R02937 | Bearded dragon (*Pogona vitticeps*) | Swab | qPCR |  | Not provided |
|  |  | 2018 | 1809S13280 | Bearded dragon (*Pogona vitticeps*) | Swab | qPCR | AdV | Not provided |
| Squamata, Chamaeleon-idae |  | 2010 | 10/10 | Jackson’s chameleon (*Trioceros jacksonii*) | Swab | nPCR |  | Died suddenly, no clinical signs. Crickets from same owner also IIV pos. |
|  |  | 2010 | 40/10 | Veiled chameleon (*Chamaeleo calyptratus*) | Intestine | CC (IgH2), nPCR |  | Obstipation (sand), cloacal prolapse |
|  |  | 2010 | 134/10 | Carpet chameleon (*Furcifer lateralis*) | Oral swab | CC (IgH2), nPCR |  | Stomatitis |
|  |  | 2011 | 126/11 | Veiled chameleon (*Chamaeleo calyptratus*) | Oral swab | CC (IgH2) |  | Enteritis |
|  |  | 2012 | 48/12 | Malagasy giant chameleon  (*Furcifer oustaleti*) | Oral swab | nPCR | AdV | Disseminated skin lesions with small pustules |
|  |  | 2012 | 71/12 | Panther chameleon (*Furcifer pardalis*) | Skint | nPCR |  | Papillomatosis |
|  |  | 2012 | 117/12 | Jackson’s chameleon (*Trioceros jacksonii*) | Liver | CC (IgH2), nPCR | AdV | Not provided |
|  |  | 2012 | 135/12 | Panther chameleon (*Furcifer pardalis*) | Oral swab | CC (IgH2), nPCR |  | CNS signs |
|  |  | 2015 | 1502T15428 | Panther chameleon (*Furcifer pardalis*) | Cloacal swab | qPCR |  | Not provided |
|  |  | 2015 | 1502T15429 | Panther chameleon (*Furcifer pardalis*) | Cloacal swab | qPCR |  | Not provided |
|  |  | 2015 | 1502T15431 | Panther chameleon (*Furcifer pardalis*) | Cloacal swab | qPCR |  | Not provided |
|  |  | 2015 | 1504S45714 | Chameleon | Tissues | qPCR |  | Not provided |
|  |  | 2015 | 1504S45715 | Chameleon | Feces | qPCR |  | Not provided |
|  |  | 2018 | 1803S23811 | Panther chameleon (*Furcifer pardalis*) | Tissues | qPCR |  | Cheilitis |
|  |  | 2018 | 1803S23812 | Panther chameleon (*Furcifer pardalis*) | Tissues | qPCR |  | Cheilitis |
|  |  | 2018 | 1803S23813 | Panther chameleon (*Furcifer pardalis*) | Tissues | qPCR |  | Cheilitis |
| Squamata, Crotaphytidae |  | 2016 | 1602S50121 | Collard lizard (*Crotaphytus collaris*) | Swab and skin | qPCR |  | Not provided |
| Squamata, Varanidae |  | 2011 | 106/22/11 | Ridgetail monitor (*Varaus acanthurus*) | Oral swab | CC (IgH2) |  | Not provided |
|  |  | 2011 | 106/23/11 | Ridgetail monitor (*Varaus acanthurus*) | Cloacal swab | CC (IgH2) |  | Not provided |
|  |  | 2011 | 106/26/11 | Ridgetail monitor (*Varaus acanthurus*) | Swab | CC (IgH2) |  | Not provided |
|  |  | 2012 | 29/12 and 64/12 | Asian water monitor (*Varanus salvator*) | GI tract, head, lung and heart | nPCR | AdV | Not provided |
| Squamata, Lacertidae |  | 2010 | 159/10 | Western green lizard (*Lacerta bilineata*) | Liver | nPCR |  | 7 of 11 hatchlings died with anorexia, dyspnea. One parent had a papillom on the skin. Possible inclusion bodies seen in liver on histology |
| Squamata, Eublepharidae |  | 2010 | 97/2/10 | Leopard gecko (*Eublepharis macularius*) | Oral and cloacal swab | CC (IgH2) |  | Obstipation (sand) |
|  |  | 2010 | 97/3/10 | Leopard gecko (*Eublepharis macularius*) | Oral and cloacal swab | CC (IgH2) |  | Wound dehiscence |
|  |  | 2011 | 120/11 | Leopard gecko (*Eublepharis macularius*) | Cloacal swab | CC (IgH2), nPCR |  | Cachexia, apathy |
|  |  | 2018 | 1809S49561 | Leopard gecko (*Eublepharis macularius*) | Swab | qPCR | Reovirus | Not provided |
| Squamata, Pythonidae |  | 2011 | 1/1/11 | Python | Oral swab | nPCR | AdV | Not provided |
|  |  | 2011 | 1/2/11 | Python | Oral swab, lung kidney | nPCR | AdV | Not provided |
|  |  | 2013 | 38/13 | Indian rock python (*Python molurus*) | Lung and heart, brain, kidney | nPCR |  | Apathy, anorexia, low weight, nasal discharge |
| Squamata, Boidae |  | 2013 | 24/9/13 | Anaconda  (*Eunectes* spp.) | Oral swab | CC (VH2), nPCR |  | Clinically healthy |
|  |  | 2013 | 24/10/13 | Anaconda (*Eunectes* spp.) | Oral swab | CC (VH2) |  | Clinically healthy |
| Squamata, species unknown |  | 2015 | 1503S39936 | Lizard | Swab | qPCR |  | Not provided |
|  |  | 2015 | 1503S39937 | Lizard | Swab | qPCR |  | Not provided |
|  |  | 2015 | 1503S39938 | Lizard | Swab | qPCR |  | Not provided |
|  |  | 2015 | 1503S39939 | Lizard | Swab | qPCR |  | Not provided |
|  |  | 2015 | 1503S39943 | Lizard | Feces | qPCR |  | Not provided |
|  |  | 2015 | 1510S86707 | Lizard | Swab | qPCR |  | Not provided |
| Anura, Bufonidae |  | 2011 | 106/21/11 | Asian common toad (*Duttaphrynus melanostictus*) | Skin swab | CC (IgH2) |  | Not provided |
| Anura, Bombinatoridae |  | 2011 | 132/2/11 | Oriental fire-bellied toad (*Bombina orientalis*) | Liver | CC (IgH2) |  | Not provided |
| Urodela, Ambystomidae |  | 2018 | 1809S23002 | Axlotl (Ambystoma mexicanum | Swab | qPCR |  | Not provided |
| Orthoptera, Gryllidae |  | 2010 | 11/10 | Crickets | 4 animals (out of 6 tested) | CC (IgH2), nPCR |  | Chameleons fed with crickets from this owner died suddenly |
|  |  | 2010 | 89/2/10 | House cricket (*Acheta domestica*) |  | CC (IgH2), nPCR |  | No pathology reported |
|  |  | 2010 | 99/10 | House cricket (*Acheta domestica*) |  | nPCR |  | Approx. 60% died, low growth, twitching, dorsal recumbancy |
|  |  | 2011 | 110/11 | Cricket |  | CC (IgH2), nPCR |  | Fed to IIV positive frogs |
|  |  | 2012 | 95/12 | Cricket |  | nPCR |  | Not provided |
|  |  | 2014 | 1406S74334 | House cricket (*Acheta domestica*) |  | qPCR |  | Not provided |
|  |  | 2015 | 1503S39929 | Insects |  | qPCR |  | Not provided |
|  |  | 2015 | 1506S45016 | Crickets |  | qPCR |  | Died suddenly |
|  |  | 2017 | 1704S02708 | Grasshopper (*Prionotropis rhodanica*) |  | qPCR |  | Not provided |
|  |  | 2018 | 1805S51467 | Cricket |  | qPCR |  | Not provided |
|  |  | 2018 | 1805S51493 | Cricket |  | qPCR |  | Not provided |
|  |  | 2018 | 1807T06279 and 1807T06291 | Grasshopper (*Prionotropis rhodanica*) |  | qPCR |  | Not provided |
|  |  | 2018 | 1807T06292 | Grasshopper (*Prionotropis rhodanica*) |  | qPCR |  | Not provided |
| Orthoptera, species unknown |  | 2015 | 1503S39929 | Insects |  | qPCR |  | Not provided |

^1^Detection methods: CC: isolation in cell culture (IgH2: iguana heart cells; BC: bearded dragon embryo cells; VH2: viper heart cells)

^2^Other viruses detected: AdV: adenovirus, detected by PCR according to Wellehan et al., 2004; Ranavirus detected by PCR according to Marschang et al., 1999; Reovirus detected by PCR according to Wellehan et al., 2008

**Literature cited:**

Marschang RE, Becher P, Posthaus H, Wild P, Thiel H-J, Müller-Doblies U, Kaleta EF, Bacciarini LC. 1999. Isolation and characterization of an iridovirus from Hermann’s tortoises (*Testudo hermanni*). Archives of Virology **144**: 1909-1922.

Wellehan JFX, Childress Al, Marschang RE, Johnson AJ, Lamirand EW, Roberts JE, Vickers ML, Gaskin JM, Jacobson ER. 2008. Consensus nested PCR amplification and sequencing of diverse reptilian, avian, and mammalian Orthoreoviruses. Vet Microbiol. **133**: 34-42.

Wellehan JF, Johnson AJ, Harrach B, Benkö M, Pessier AP, Johnson CM, Garner MM, Childress A, Jacobson ER. 2004 Detection and analysis of six lizard adenoviruses by consensus primer PCR provides further evidence of a reptilian origin for the atadenoviruses. J Virol. 7(23):13366-9.
