# Supplementary material for: Detection and Characterization of Invertebrate Iridoviruses Found in Reptiles and Prey Insects in Europe over the Past Two Decades
Source: Viruses. 2019 Jul 2;11(7):600. doi: 10.3390/v11070600 (PMC6669658; doi:10.3390/v11070600)
Supplement: Supplementary file 1 [file viruses-11-00600-s001.zip › IIV_Supplementary Table S4-sequence identities_final.docx]

**Supplementary Table S4.** Sequence identity values (%) between Liz-CrIV and IIV6

| Region: | Exonuc. | Polym. | ATPase | Viral Antibiotic Peptide and flanking genes | | | | | Ligase | Thym. synt. | Major Capsid Protein and flanking genes | | | | I.E. prot. |
| --- | --- | --- | --- | --- | --- | --- | --- | --- | --- | --- | --- | --- | --- | --- | --- |
| ORF (IIV6) | 012L | 037L | 075L | 155L | 157L | 159L | 160L | 161L | 205L | 225R | WIV orf011 | 274L | 281R | 282R | 393L |
| **Length** (nt)  full/partial | 1959  full | 3831  full | 650  part | 1267  full | 459  full | 930  full | 198  full | 150  part. | 1293  part. | 507  part. | 549  na | 1428  full | 144  full | 377  part. | 672  part. |
| DNA | 91.2 | 97.0 | 97.8 | 97.4 | 95.4 | 54.5* | 73.6 | 99.3 | 95.8 | 95.7 | 43.8* | 94.5 | 84.9 | 98.1 | 97.8 |
| PROTEIN | 91.6 | 96.7 | 100 | 99.2 | 98.0 | 46.0* | 39.6 | 100 | 97.2 | 92.4 | NA | 95.5 | 78.8 | 97.6 | 96.3 |

*Long gaps were omitted in the comparison to maximise identity values. NA= not applicable
