# Supplementary material for: Detection and Characterization of Invertebrate Iridoviruses Found in Reptiles and Prey Insects in Europe over the Past Two Decades
Source: Viruses. 2019 Jul 2;11(7):600. doi: 10.3390/v11070600 (PMC6669658; doi:10.3390/v11070600)
Supplement: Supplementary file 1 [file viruses-11-00600-s001.zip › IIV_Supplementary_Table-S2-primers.docx]

### Supplementary Table S2 (continue next page) Primers used for sequence comparison of isolates

| **Primer name** | **5’ – Sequence – 3’** | **Reference** |
| --- | --- | --- |
| MCP-F1 | GGTTTCATCGATATCGCCAC | Jakob et al., 2002 [45] |
| MCP-F3 | GGGCCGGAGATTATTTGTT |  |
| MCP-R3 | GACAATAGCATAGTTACCCCA |  |
| MCP-R4 | GAAAAGTAATCACTGCCCAT |  |
| 012L-R1 | ATATGACCATTCCCAATCTGGAAC |  |
| 012L-F1 | GGTTGATGATACAGCAGAATGGTC |  |
| 037L-R1 | TGATCCTAAAAAATGTTTCGCCAC |  |
| 037L-F1 | GAAGGAAGAGGAGCGTTAGCT |  |
| 075L-R1 | TCATGGCATCCAAATTTCATATCTC |  |
| 075L-F1 | GTCAGACACACAAGAATATAGCAT |  |
| 205R-R1 | CTACAATTCAGCAATCTCCTTTCC |  |
| 205R-F1 | TATTTCATCAAGGACAAATGTTTACC |  |
| 393L-R1 | TTGAACCATTCATGTAATTCAAGCAA |  |
| 393L-F1 | ATAAGTGGATTATTACAACTCATCGA |  |
| IIV-012L 1010F | CAGATAACAATGGTTTGGGT | this study |
| IIV-012L 1036F | TCTTGTCAGGACGTAGTCTT |  |
| IIV-012L 1642R | AATCAGAACTATCTGGAGGA |  |
| IIV-012L 274F | GGAGTAGCTCCAAGAAGTAA |  |
| IIV-012L 938R | GGAATATGTGGCAAGAAATC |  |
| IIV-012L 262R | AACTGCAACTGCAACATC |  |
| IIV-012L 436R | GGTTTGTTCTTCTACACG |  |
| IIV-012L 975F | TGCAACTTCGTCATCATC |  |
| IIV-012L 39F | TGTTCAATTCCGTGAAG |  |
| IIV-012L 77F | ACAAGTTGGACGTCTTCTAG |  |
| IIV-012L 744R | GCATAAATTCGGTTCCTG |  |
| IIV-012L -237F | TCTAGCAGAAGCTTTGTGT |  |
| IIV-012L 265R | GGAGCTACTCCGTCGATAC |  |
| IIV-012L 501R | TTTGTGGAGCATCGGTNGT |  |
| IIV-012L1582F | GCAACTTCGTCATCATCC |  |
| IIV-012L 2196R | GGAACTGCAACTGCAACA |  |
| IIV-012L -218F | TCAGAGAAACGTTCTTCA |  |
| IIV-012L -27F | AGTTAAAGAAGATTAGTA |  |
| IIV-012L 239R | TTGTATAACCATTCTGTC |  |
| IIV-012L -1250F | GCTTTAACAGAACTTGCTGC |  |
| IIV-012L 285R | TGGAGCTACTCCGTCGATACA |  |
| IIV-012L 1532F | ACGACTGAGCCCCAGGAAAC |  |
| IIV-012L 2863R | GTGGCGTTAAAACAACCCGTT |  |
| IIV-037L 1577F | AATTATAGAGGTGAAGTGTT |  |
| IIV-037L 2241R | TTCTTATATGTTCTTGTTCC |  |
| IIV-037L 2455R | AACACACATTACACTCTTAC |  |
| IIV-037L -12F | GTACATTATAAAATGGAG |  |
| IIV-037L 715R | TTACCATCTTCAGAATATAC |  |
| IIV-037L 1199F | CATATGGAACAACAGATT |  |
| IIV-037L 1737R | GTTGACCATGAACATGTA |  |
| IIV-037L 1639F | AGTGTTTTGGTAGCCGACAT |  |
| IIV-037L 2742R | TGAATCGGTATCCCCATACA |  |
| IIV-037L 3418R | CTTGACAAGGCAGTCGTTCT |  |
| IIV-037L 2025F | ATGGGATGATCATGTTGCTTGT |  |
| IIV-037L 2970F | GCGGAAAGGATGGAAATGTT |  |

**Suppl. Table 2 (continued)**

| **Primer name** | **5’ – Sequence – 3’** | **Reference** |
| --- | --- | --- |
| IIV-037L 4084R | CTCATGGTCCTGCTTGGTCA | this study |
| IIV-149L 61452F | AAGCATAGTAGAATCGTTTGCG |  |
| IIV-155L 62425F | GCTTGTCTAAATTGAGGATCGG |  |
| IIV-155L 62625R | CAGTGTAGATAATCATGTATTC |  |
| IIV-157L Rev1 | GATCTAATTTAAATGTATCC |  |
| IIV-157L Rev2 | GACTTTATCGATTTCCATTG |  |
| IIV-158L 63444F | CGATTTCCATTGTAGATACAATG |  |
| IIV-158L 63466R | CATTGTATCTACAATGGAAATCG |  |
| IIV-159L Fwd1 | CTGAAACGGTTATTACATGG |  |
| IIV-159L Fwd2 | CAAATGCTACATCTGCAGCTGG |  |
| IIV-159L 64726R | CWATTACWGCTGGAGAATTAGC |  |
| IIV-159L 64757F | CCGTAATTGTTCCTGCTGC |  |
| IIV-159L 64795F | CCAGTAATTGTTCCTGCTGCG |  |
| IIV-159L 65421F | CATTATTGTTAGCAGCGGATG |  |
| IIV-160L 65107F | GCACAATTTCCAGAACAACAACC |  |
| IIV-162L 66728R | CGTATACGGTTTACACTCCAC |  |
| MCP 127908F | GGACAAGAAGAAAGCGCTTCTAC |  |
| MCP 129276R | CCTAACTACGATATTCGCTTCTCTC |  |
| MCP 128609F | GTAACAGAAAGTGGATATGATATAG |  |
| MCP 128918R | GTATTGTACCAACTGCAAGCC |  |
| MCP 129980F | GTTAGCTGATCCGTTTGCTCTAG |  |
| MCP 130721R | GTTCTGTAAGATATGATTTTGCTAC |  |
| MCP 127908F | GGACAAGAAGAAAGCGCTTCTAC |  |

**Cited literature:**

Jakob, N.J.; Kleespies, R.G.; Tidona, C.A.; Müller, K.; Gelderblom, H.R.; Darai, G. Comparative analysis of the genome and host range characteristics of two insect iridoviruses: Chilo iridescent virus and a cricket iridovirus isolate. *J Gen Virol* **2002**, *83*, 463-470, doi:10.1099/0022-1317-83-2-463.
