# Supplementary material for: Detection and Characterization of Invertebrate Iridoviruses Found in Reptiles and Prey Insects in Europe over the Past Two Decades
Source: Viruses. 2019 Jul 2;11(7):600. doi: 10.3390/v11070600 (PMC6669658; doi:10.3390/v11070600)
Supplement: Supplementary file 1 [file viruses-11-00600-s001.zip › IIV_Suppl_Table S1_Bearded dragons.docx]

### Supplementary Table S1. Bearded dragon transmission study basic data

| **Animal** | **Sex** | **Survival (60 dpi)** | **weight at**  **(g)** | | **length at**  **(mm)** | | **Coelomic inocoluate (ml)** | **Gastric inoculate (ml)** |
| --- | --- | --- | --- | --- | --- | --- | --- | --- |
|  |  |  | **begin** | **end.** | **begin** | **end.** |  |  |
| NK1 | ♂ | living | 30,6 | 43,3 | 95,0 | 106,5 | 1 | 1 |
| NK2 | ♀ | living | 37,3 | 46,9 | 97,2 | 108,5 | 1 | 1 |
| NK3 | ♂ | living | 11,4 | 16,1 | 67,5 | 75,5 | 1 | 1 |
| NK4 | ♀ | living | 17,1 | 21,0 | 84,0 | 88,3 | 1 | 1 |
| NK5 | ♂ | living | 11,5 | 13,6 | 66,8 | 70,6 | 1 | 1 |
| A1 | ♀ | living | 23,4 | 26,6 | 85,3 | 91,0 | — | 1 |
| A2 | ♀ | living | 25,0 | 31,4 | 92,2 | 92,7 | — | 1 |
| A3 | ♀ | living | 9,8 | 12,6 | 64,5 | 69,8 | — | 1 |
| A4 | ♀ | living | 12,0 | 16,2 | 71,0 | 76,8 | — | 1 |
| A5 | ♂ | † 16 days | 5,7 | 5,1 | 53,0 | 53,0 | — | 0,8 |
| B1 | ♀ | living | 28,1 | 36,4 | 96,5 | 107,2 | 1 | 1 |
| B2 | ♀ | living | 10,8 | 12,2 | 62,7 | 65,4 | 1 | 0,9 |
| B3 | **♀** | living | 8,7 | 9,3 | 57,6 | 59,8 | 1 | 1 |
| B4 | ♂ | living | 18,9 | 25,3 | 85,2 | 92,0 | 1 | 0,9 |
| B5 | ♂ | living | 23,8 | 27,2 | 88,7 | 91,9 | 1 | 1 |
