# Supplementary material for: Detection and Characterization of Invertebrate Iridoviruses Found in Reptiles and Prey Insects in Europe over the Past Two Decades
Source: Viruses. 2019 Jul 2;11(7):600. doi: 10.3390/v11070600 (PMC6669658; doi:10.3390/v11070600)
Supplement: Supplementary file 1 [file viruses-11-00600-s001.zip › Suppl Fig S2_final.docx]

**CIV-VAB**  ATGTCGATATTACTTAAAATTTTATTTAAATTGTTGTTATTGATCTTATCTATTACATTTGTAATAACAGATTGTTTGCCTAGAAGCTGTGCTTCTTTTG 100

**CrIV-var** ................................AC........................C.........**-------**......................... 93

**CIV-VAB**  GTTGTTGTTCTGGAAATTGTGCAACCTGGTGTGATCAATGCGATATTAAATATTCATGTTAA 162

**CrIV-var** .........................T....................................AACTTCTTCTATGTCTCCCTTTTTATACAGGAAGATAAGGTAA 198

**CIV-VAB**  MSILLKILFKLLLLILSITFVITDCLPR--------SCASFGCCSGNCATWCDQCDIK---YSC 53

**CrIV-var** ...................S...-..EAVLLLVVVLEIVQL.VINAILNIHVKTSSMSPFL.RKIR 65

3600

3400

3200

3000

2800

2600

2400

2200

2000

1800

1600

1400

1200

1000

800

600

400

200

0

**1.0**

0.9

0.8

0.7

0.6

**0.5**

0.4

0.3

0.2

0.1

0.0

**Position**

**S i m i l a r i t y**

ORF161L

IIV6 ORF159L start / WIV ORFs

IIV6 ORF157L

IIV6 ORF155L & ORF149L start

Window: 200 bp, Step: 20 bp,

Jukes-Cantor (1-parameter)

SimPlot - Query: **GbIV-variants IIV6-VAB like & flankings**

**Legend:**

IIV6 genome [AF303741] part 61.5‒65.4 kb (ORF 149L‒161L)

IIV9 genome [GQ918152] part 9.9‒13.3 kb (ORF 012R‒015R)

IIV9 genome [GQ918152] part 35.6‒39 kb (ORF 038L‒040R)

**VAB**

VAB?

**Seuppl. Fig. S2.** SimPlot analysis of the VAB homologue gene and its flanking region from our IIV isolates. The corresponding region of IIV6 along with two partially homologeous sections of IIV9 were included in the alignment. Gene homologs with the highest BLAST values are drawn under the diagram. The third ORF (910 nt) shows a suspected recombination site. Alignment of the IIV6-VAB with that of these isolates is shown above the graph.
