# Supplementary material for: Detection and Characterization of Invertebrate Iridoviruses Found in Reptiles and Prey Insects in Europe over the Past Two Decades
Source: Viruses. 2019 Jul 2;11(7):600. doi: 10.3390/v11070600 (PMC6669658; doi:10.3390/v11070600)
Supplement: Supplementary file 1 [file viruses-11-00600-s001.zip › Suppl Fig S3.docx]

0.1

IIV22

BIV

SGIV

LCDV-C

SDDV

SERV

CoIV

IIV30

IIV9 (WIV)

GSIV

EHNV

ADRV

DFV

**Liz-Cr IV**

TRBIV

GGRV

CH8-96

IIV22a

IIV3

LCDV1

ESV

Rmax

ECV

ToRV1

ATV

FV3

CMTV

LMBV

LYCIV

PPIV

IIV6 (CIV)

RSIV

TFV

OSGIV

RBIV

STIV

RGV

LCDV-Sa

GIV

IIV25

CMTV

AMIV

IIV31

GV6

ISKNV

SSME

*1*

*1*

1

*1*

*1*

1

*0,79*

1

*1*

*1*

*1*

1

*1*

*1*

1

*1*

1

1

*1*

*1*

*1*

1

1

0,96

1

*0,79*

*1*

1

*0,81*

*1*

*1*

1

1

*1*

*1*

*1*

*1*

*0,83*

*1*

*1*

*1*

*1*

***Chloridovirus***

***Megalocytivirus***

***Ranavirus***

***Lymphocystivirus***

***Iridovirus***

*1*

**Supplementary Figure S3**

Phylogenetic tree reconstruction of *Iridoviridae*. Bayesian tree was constructed using the deduced amino acid (aa) alignments of 25 core genes (in case of Liz-CrIV and IIV6: 337L, 176R, 022L, 369L, 274L, 347L, 143R, 436R, 349L, 142R, 376L, 428L, 037L, 098L, 118L, 179R, 355R, 295L, 184R, 067R, 380R, 287R, 282R), selecting Whelan & Goldman with gamma distribution (WAG+G) model. Two independent runs were performed for 10^6^ generations. Every tenth tree was sampled out of which 25% was discarded as burn-in.

Liz-CrIV sequence was added and realigned to the alignment available from the online database of the International Committee on the Taxonomy of Viruses (ICTV): <https://talk.ictvonline.org/ictv-reports/ictv_online_report/dsdna-viruses/w/iridoviridae/609/resources-iridoviridae>

**IIV6/CIV**: Chilo iridescent virus; **IIV3**: Aedes taeniorhynchus iridescent virus; **ATV**: Ambystoma tigrinum stebbensi virus; **TFV**: Tiger frog virus;

**SGIV**: Singapore grouper iridovirus; **GIV**, Grouper iridovirus; **STIV**: Soft shelled turtle iridovirus; **LCDV-C**: Lymphocystis disease virus isolate China;

**LCDV1**: Lymphocystis disease virus 1; **ISKNV**: Infectious spleen and kidney necrosis virus; **RBIV**: Rock bream iridovirus;

**OSGIV**: Orange spotted grouper iridovirus; **EHNV**: Epizootic hematopoietic necrosis virus
