# Supplementary material for: Detection and Characterization of Invertebrate Iridoviruses Found in Reptiles and Prey Insects in Europe over the Past Two Decades
Source: Viruses. 2019 Jul 2;11(7):600. doi: 10.3390/v11070600 (PMC6669658; doi:10.3390/v11070600)
Supplement: Supplementary file 1 [file viruses-11-00600-s001.zip › Suppl Fig S5_Bearded dragon transmission study_final_corr.docx]

**Suppl. Figure S5 (continue next 5 pages):**  All of the figure segments on the following pages are divided into a top part with a graph and table showing the qPCR values recalculated for 20 mg tissue starting material for DNA extraction, and in the table below the nPCR and virus isolation results are shown. (+) indicates a weak band for nPCR and inconsistent results (contradicting in the repeats) for virus isolation.

**Negative control bearded dragons:**

**lizard BD-NK1 organs:**

0

10

20

30

40

mean copy number

0,76625

0,63727

2,48949

2,15602

2,85199

2,19379

1,27941

2,19119

0,45466

1,55341

2,10567

0,18774

mean Ct value

35,6039

35,8151

34,8666

34,9817

34,0978

34,9681

35,0164

34,3998

36,2021

34,7941

34,0001

37,2157

brain

gonad

kidney

skin

lung

heart

fat body

liver

tongue

gaster

ileum

colon

mean copy number

mean Ct value

| nPCR | — | — | — | — | — | — | — | — | — | — | — | — |
| --- | --- | --- | --- | --- | --- | --- | --- | --- | --- | --- | --- | --- |
| cell culture | — | — | — | — | — | — | — | — | — | — | — | (+) |

**lizard BD-NK2 organs:**

0

10

20

30

40

mean copy number

mean Ct value

mean copy number

1,75663

1,91847

1,4372

1,97137

1,78372

0,46328

3,37834

0,59345

0,65534

3,23957

1,44076

0,6531

mean Ct value

34,6532

34,5522

34,8832

34,521

34,6356

36,1805

33,9037

35,8968

35,7831

33,9518

34,8803

35,787

brain

gonad

kidney

skin

lung

heart

fat body

liver

tongue

gaster

ileum

colon

| nPCR | — | — | — | — | — | — | — | — | — | — | — | — |
| --- | --- | --- | --- | --- | --- | --- | --- | --- | --- | --- | --- | --- |
| cell culture | — | — | — | — | — | — | — | — | — | — | — | (+) |

**lizard BD-NK3 organs:**

0

10

20

30

40

mean copy number

0,02996

undet.

3,09918

1,49828

0,15751

0,10863

1,02363

0,52267

2,52776

0,25996

0,5588

0,20852

mean Ct value

37,8296

32,9869

33,7456

36,0971

36,485

34,1433

34,845

33,1996

35,5741

34,7752

35,8043

brain

gonad

kidney

skin

lung

heart

fat body

liver

tongue

gaster

ileum

colon

>40

mean copy number

mean Ct values

| nPCR | — | — | — | — | — | — | — | — | — | — | — | — |
| --- | --- | --- | --- | --- | --- | --- | --- | --- | --- | --- | --- | --- |
| cell culture | — | — | — | — | — | — | — | — | — | — | — | — |

**lizard BD-NK4 organs:**

0

10

20

30

40

mean copy number

0,57156

0,13041

0,51243

0,2958

0,18352

0,45125

0,76044

0,30985

0,34934

0,15442

0,15029

0,76256

mean Ct values

34,7516

36,2942

34,8657

35,4393

35,9376

34,9984

34,4536

35,3908

35,2656

36,1178

36,1461

34,4507

brain

gonad

kidney

skin

lung

heart

fat body

liver

tongue

gaster

ileum

colon

mean copy number

mean Ct values

| nPCR | — | — | — | — | — | — | — | — | — | — | — | — |
| --- | --- | --- | --- | --- | --- | --- | --- | --- | --- | --- | --- | --- |
| cell culture | — | — | — | — | — | — | — | — | — | — | — | — |

**lizard BD-NK5 organs:**

0

10

20

30

40

mean copy number

mean Ct value

mean copy number

0,11157

undet.

0,00597

0,19392

0,17075

0,1356

0,78455

0,25798

undet.

0,21599

undet.

undet.

mean Ct value

37,1425

36,402

36,4748

37,0259

34,3605

36,0581

36,1586

brain

gonad

kidney

skin

lung

heart

fat body

liver

tongue

gaster

ileum

colon

>40

>40

>40

>40

39,5816

| nPCR | — | — | — | — | — | — | — | — | — | — | — | — |
| --- | --- | --- | --- | --- | --- | --- | --- | --- | --- | --- | --- | --- |
| cell culture | — | — | — | — | — | — | — | — | — | — | — | — |

**“group A” infected” bearded dragons:**

Please note that the qPCR graphs have two different Y-axis scaling for the mean copy number /logarithmic/ and the mean Ct values /linear/.

**lizard BD-A1 organs:**

0,1

10

1000

100000

10000000

0

5

10

15

20

25

30

35

mean copy numbers

17,33155

71,33109

11,38026

2431,141

7,027988

54,42955

0,895851

1,334234

138,9915

3,548637

0,77282

122223,5

mean Ct values

29,87605

28,94312

30,41985

22,58908

30,14686

28,39664

32,8098

32,29485

26,28859

31,24035

33,00078

18,42077

brain

gonad

kidney

skin

lung

heart

fat body

liver

tongue

gaster

ileum

colon

mean copy numbers

mean Ct values

1

| nPCR | + | + | + | + | + | + | — | — | + | + | — | + |
| --- | --- | --- | --- | --- | --- | --- | --- | --- | --- | --- | --- | --- |
| cell culture | — | — | — | + | — | (+) | — | — | (+) | — | — | + |

**lizard BD-A2 organs:**

0,01

1

100

10000

1000000

0

5

10

15

20

25

30

35

40

mean copy number

10,53486

12,51816

0,535421

40,21178

0,934988

5,629609

0,736848

0,082606

766,2797

255,4266

127,7133

16194,93

mean Ct values

31,54414

35,19417

36,29218

29,8124

33,77917

32,3543

34,08707

36,78743

26,00187

35,53264

33,81247

21,16136

brain

gonad

kidney

skin

lung

heart

fat body

liver

tongue

gaster

ileum

colon

mean copy numbers

mean Ct values

| nPCR | + | — | — | + | — | — | — | — | + | + | + | + |
| --- | --- | --- | --- | --- | --- | --- | --- | --- | --- | --- | --- | --- |
| cell culture | — | — | — | — | — | — | — | — | + | + | + | + |

**lizard BD-A3 organs:**

mean copy numbers

mean Ct values

1

100

10000

1000000

0

5

10

15

20

25

30

35

40

mean copy number

836,6888

271,7185

3205,19

52269,95

24,05668

1,797944

484,032

28,12551

9,739005

267,391

56,41932

135959,2

mean Ct value

24,01612

27,99982

23,17293

20,4504

27,72127

33,41091

25,62544

27,51854

28,89444

24,73351

26,61539

16,5124

brain

gonad

kidney

skin

lung

heart

fat body

liver

tongue

gaster

ileum

colon

| nPCR | + | + | + | + | + | — | + | + | + | + | + | + |
| --- | --- | --- | --- | --- | --- | --- | --- | --- | --- | --- | --- | --- |
| cell culture | + | — | (+) | (+) | — | — | (+) | + | — | + | — | + |

1

100

10000

1000000

0

5

10

15

20

25

30

mean copy number

967,2847

801,9347

3299,681

115416,5

46,68628

541,1742

112,4863

352,8924

126,3643

1369,36

4100,515

224886,3

mean Ct value

25,53853

28,33109

24,82325

19,81701

28,51265

27,63046

26,94922

25,41759

27,72193

23,60114

22,13188

16,76738

brain

gonad

kidney

skin

lung

heart

fat body

liver

tongue

gaster

ileum

colon

**lizard BD-A4 organs:**

mean copy numbers

mean Ct values

| nPCR | + | + | + | + | + | + | + | + | + | + | + | + |
| --- | --- | --- | --- | --- | --- | --- | --- | --- | --- | --- | --- | --- |
| cell culture | + | — | (+) | + | — | (+) | — | + | — | + | + | + |

1

100

10.000

1.000.000

100.000.000

0

5

10

15

20

25

30

35

Quantity Mean

63,995

81,3966

100,488

465,979

1119,33

2790,60

135,44

6421,14

150638

111748

3108,3

3870119

13,002

mean Ct

29,4958

29,1865

28,9109

26,8958

26,2674

24,5476

28,513

23,6511

19,323

19,853

24,407

25,045

31,630

brain

gonad

kidney

skin

lung

heart

fat body

liver

tongue

gaster

ileum

colon

trachea

**lizard BD-A5 organs:**

mean copy numbers

mean Ct values

| nPCR | + | + | + | + | + | + | + | + | + | + | + | + | + |
| --- | --- | --- | --- | --- | --- | --- | --- | --- | --- | --- | --- | --- | --- |
| cell culture | (+) | (+) | (+) | (+) | (+) | (+) | (+) | + | + | + | + | + | (+) |

Please note that the qPCR graphs have two different Y-axis scaling for the mean copy number /logarithmic/ and the mean Ct values /linear/.

**“group B” infected bearded dragons:**

0,1

1

10

100

1000

10000

100000

0

5

10

15

20

25

30

35

40

45

mean copy number

2,068607

0,116842

35,93343

2108,226

5,721065

0,107455

43,07851

1,17953

9,383245

0,293217

0,628192

52041,57

mean Ct value

35,69173

38,49607

29,96328

23,51591

32,34272

39,84634

28,65036

34,45121

32,59034

34,75538

34,66544

22,09186

brain

gonad

kidney

skin

lung

heart

fat body

liver

tongue

gaster

ileum

colon

**lizard BD-B1 organs:**

mean copy numbers

mean Ct values

| nPCR | (+) | — | + | + | + | — | + | + | (+) | — | — | + |
| --- | --- | --- | --- | --- | --- | --- | --- | --- | --- | --- | --- | --- |
| cell culture | — | — | — | + | — | — | — | — | — | — | — | + |

1

100

10000

1000000

0

5

10

15

20

25

30

35

mean copy number

3685,439

18639,22

11430,03

30816,71

84,84039

397,8712

484,1405

2516,179

57,51813

1604,407

52010,9

467787,1

mean Ct values

23,20341

22,88915

22,91463

20,90166

28,7988

26,18543

25,37937

22,78612

28,23314

23,26125

19,65734

15,78621

brain

gonad

kidney

skin

lung

heart

fat body

liver

tongue

gaster

ileum

colon

**lizard BD-B2 organs:**

mean copy numbers

mean Ct values

| nPCR | + | + | + | + | + | + | + | + | + | + | + | + |
| --- | --- | --- | --- | --- | --- | --- | --- | --- | --- | --- | --- | --- |
| cell culture | (+) | (+) | (+) | + | — | (+) | (+) | (+) | — | (+) | (+) | + |

0,01

1

100

10000

1000000

100000000

0

5

10

15

20

25

30

35

40

45

mean copy number

157,0325

11753,08

0,07687

410447,3

115,2874

256,9836

17496,4

136,2962

430,2419

236,6456

1,387705

2195139

mean Ct value

28,07127

26,44616

39,56895

17,52804

27,79838

29,86099

23,97426

27,83133

28,53725

28,6946

33,89452

16,73113

brain

gonad

kidney

skin

lung

heart

fat body

liver

tongue

gaster

ileum

colon

**lizard BD-B3 organs:**

mean copy numbers

mean Ct values

| nPCR | + | — | — | + | + | + | + | + | + | + | (+) | + |
| --- | --- | --- | --- | --- | --- | --- | --- | --- | --- | --- | --- | --- |
| cell culture | + | (+) | — | + | (+) | — | — | (+) | + | + | — | + |

0,1

10

1000

100000

0

5

10

15

20

25

30

35

40

45

mean copy number

9,187306

2,44995

3,948333

1738,796

1,120712

1,665637

0,148484

1,743048

0,581596

2,156099

502,7678

23303,19

mean Ct value

33,10958

36,03004

32,60687

25,43336

32,80478

35,37685

38,17025

32,7602

34,31198

32,69844

25,98483

20,94325

brain

gonad

kidney

skin

lung

heart

fat body

liver

tongue

gaster

ileum

colon

**lizard BD-B4 organs:**

mean copy numbers

mean Ct values

| nPCR | — | (+) | (+) | + | + | — | — | + | (+) | + | + | + |
| --- | --- | --- | --- | --- | --- | --- | --- | --- | --- | --- | --- | --- |
| cell culture | — | — | — | + | — | — | — | — | — | — | + | + |

0,1

1

10

100

1000

0

5

10

15

20

25

30

35

40

mean copy number

43,81467

0,818896

0,322029

935,0723

0,755868

0,707631

2,167637

0,85326

84,44505

0,389903

0,65162

448,0827

mean Ct value

30,43359

36,94032

36,10771

25,87417

34,64267

35,22318

33,34531

33,14054

27,98163

35,45784

33,97185

25,4272

brain

gonad

kidney

skin

lung

heart

fat body

liver

tongue

gaster

ileum

colon

**lizard BD-B5 organs:**

mean copy numbers

mean Ct values

| nPCR | + | — | — | + | (+) | — | + | + | + | — | — | + |
| --- | --- | --- | --- | --- | --- | --- | --- | --- | --- | --- | --- | --- |
| cell culture | — | — | — | (+) | — | — | — | — | (+) | — | — | + |
