# Supplementary material for: Detection and Characterization of Invertebrate Iridoviruses Found in Reptiles and Prey Insects in Europe over the Past Two Decades
Source: Viruses. 2019 Jul 2;11(7):600. doi: 10.3390/v11070600 (PMC6669658; doi:10.3390/v11070600)
Supplement: Supplementary file 1 [file viruses-11-00600-s001.zip › Suppl-FigS4_cricket bioassay_final.docx]

**Supplementary Figure S4 (continue on next five pages):**  The figure sections on the following pages have a top part showing a graph and a table showing the qPCR values recalculated for 20 mg tissue starting material for DNA extraction, and a lower table showing the evaluation of the qPCR, and the results of the nPCR and virus isolation.

The qPCR values for calculated copy numbers per µl template DNA were coded as follows:

(+)= 1- 5 copies/µl, += 5-100 copies/µl. ++= 1xE2-1xE4 copies/µl, +++= 1xE4-1xE7 copies/µl, ++++= over 1xE7 copies/µl

For nPCR, results were interpreted based on intensity of the second round MCP gene PCR products by electrophoresis, (+) =faint, += normal bands.

For virus isolation (+) means inconsistent results (contradictions in the repeats).

On the “survival” line, numbers indicate day of death post infection, or if marked with a star (*), then the day of euthanasia for “humane” reasons as the cricket was in a sublethal state. Smilies indicate survival until the end of study, (*) a sublethal state is marked with star. Abbreviations for ages: ad=adult, ny=nymph (last stage instar), la=larva (6^th^ or 7^th^ stage instar), ecd= in state of ecdysis, star (*) indicates visible malformation as shown on Figure 5 in the paper.

Mean copy No

0,001

1

1E+3

1E+6

1E+9

0

5

10

15

20

25

30

35

40

45

0,159

2E+07

0,078

0,176

0,248

0,096

0,74

6E+07

14,53

0,099

0,194

1,852

0,005

0,841

1,355

0,142

5E+07

15,94

5,369

1,142

Mean Ct value

34,74

10,44

35,64

34,61

34

35,04

33,84

8,663

31,76

35,48

34,49

31,65

39,01

33,3

32,47

34,89

9,988

28,93

30,57

32,26

Inf 1

Inf 2

Inf 3

Inf 4

Inf 5

Inf 6

Inf 7

Inf 8

Inf 9

Inf 10

Inf 11

Inf 12

Inf 13

Inf 14

Inf 15

Inf 16

Inf 17

Inf 18

Inf 19

Inf 20

**Cricket infection with lizard isolate at 20 ºC**

**:**

mean copy numbers

mean Ct values

| **qPCR** | | **—** | **++++** | **—** | **—** | **—** | **—** | **—** | **++++** | **+** | **—** | **—** | **(+)** | **—** | **—** | **(+)** | **—** | **++++** | **+** | **+** | **(+)** |
| --- | --- | --- | --- | --- | --- | --- | --- | --- | --- | --- | --- | --- | --- | --- | --- | --- | --- | --- | --- | --- | --- |
| **nPCR** | | **—** | **+** | **—** | **—** | **—** | **—** | **—** | **+** | **+** | **—** | **—** | **(+)** | **—** | **(+)** | **(+)** | **—** | **+** | **+** | **+** | **(+)** |
| **Isolation** | | **—** | **+** | **—** | **—** | **—** | **—** | **—** | **+** | **—** | **—** | **—** | **—** | **—** | **—** | **—** | **—** | **+** | **(+)** | **—** | **—** |
| **Iridescence** | | **—** | **+** | **—** | **—** | **—** | **—** | **—** | **+** | **—** | **—** | **—** | **—** | **—** | **—** | **—** | **—** | **+** | **—** | **—** | **—** |
| **Survival** (dpi) | | **☺** | **33** | **☺** | **☺** | **☺** | **☺** | **☺** | **35** | **55** | **☺** | **☺** | **59** | **☺** | **☺** | **59** | **☺** | **58** | **58** | **59** | **☺** |
| **Sex** | | **♂** | **♂** | **♂** | **♂** | **♂** | **♂** | **♂** | **♂** | **♂** | **♂** | **♂** | **♂** | **♂** | **♂** | **♂** | **♂** | **♂** | **♂** | **♂** | **♂** |
| **age** | **at begin** | **ny** | **ny** | **ny** | **ny** | **ny** | **ny** | **ny** | **ny** | **ny** | **ny** | **ny** | **ny** | **ny** | **ny** | **ny** | **ny** | **ny** | **ny** | **ny** | **ny** |
|  | **at end** | **ad** | **ad** | **ad** | **ad** | **ad** | **ad** | **ad** | **ad** | **ad** | **ad** | **ad** | **ad** | **ad** | **ad** | **ad** | **ad** | **ad** | **ad** | **ad** | **ad** |

26,97

29,823

32,685

9,1538

31,216

27,182

29,715

9,4215

13,902

30,199

9,6622

30,298

10,436

27,586

26,87

28,173

29,012

29,044

26,619

27,804

Mean copy No

1

100

10.000

1000.000

100.000.000

10.000.000.000

0

5

10

15

20

25

30

35

11,063

2,6823

2,9659

2E+08

3,9926

95,999

13,08

1E+08

3E+07

9,0483

1E+08

9,5178

5E+07

69,839

122,72

43,992

22,705

22,116

149,55

59,228

Inf 1

Inf 2

Inf 3

Inf 4

Inf 5

Inf 6

Inf 7

Inf 8

Inf 9

Inf 10

Inf 11

Inf 12

Inf 13

Inf 14

Inf 15

Inf 16

Inf 17

Inf 18

Inf 19

Inf 20

**Cricket infection with lizard isolate at 30 ºC, study I**

**:**

mean copy numbers

mean Ct values

Mean Ct value

| **qPCR** | | **+** | **(+)** | **(+)** | **++++** | **(+)** | **+** | **+** | **++++** | **++++** | **+** | **++++** | **+** | **++++** | **+** | **+** | **+** | **+** | **+** | **+** | **+** |
| --- | --- | --- | --- | --- | --- | --- | --- | --- | --- | --- | --- | --- | --- | --- | --- | --- | --- | --- | --- | --- | --- |
| **nPCR** | | **+** | **—** | **—** | **+** | **—** | **+** | **—** | **+** | **+** | **—** | **+** | **—** | **+** | **+** | **+** | **(+)** | **(+)** | **(+)** | **+** | **+** |
| **Isolation** | | **—** | **—** | **—** | **+** | **—** | **—** | **—** | **+** | **(+)** | **—** | **+** | **—** | **+** | **—** | **—** | **—** | **—** | **—** | **—** | **—** |
| **Iridescence** | | **—** | **—** | **—** | **+** | **—** | **—** | **—** | **+** | **—** | **—** | **+** | **—** | **+** | **—** | **—** | **—** | **—** | **—** | **—** | **—** |
| **Survival** (dpi) | | **☺** | **☺** | **35** | **26** | **☺** | **35** | **32** | **28** | **47** | **35** | **32** | **☺** | **☺** | **46** | **58** | **35** | **☺** | **☺** | **33** | **☺** |
| **Sex** | | **♂** | **♂** | **♀** | **♀** | **♂** | **♀** | **♀** | **♀** | **♀** | **♀** | **♂** | **♂** | **♀** | **♀** | **♀** | **♀** | **♂** | **♂** | **♀** | **♂** |
| **age** | **at begin** | **ad** | **ad** | **ad** | **ad** | **ad** | **ad** | **ad** | **ad** | **ad** | **ad** | **ad** | **ad** | **ad** | **ad** | **ad** | **ad** | **ad** | **ad** | **ad** | **ad** |
|  | **at end** | **ad** | **ad** | **ad** | **ad** | **ad** | **ad** | **ad** | **ad** | **ad** | **ad** | **ad** | **ad** | **ad** | **ad** | **ad** | **ad** | **ad** | **ad** | **ad** | **ad** |

Mean copy No

0,001

1

1000

1000000

1000000000

0

5

10

15

20

25

30

35

40

45

0,153

0,265

0,779

8,27

0,131

0,162

0,227

8E+08

0,233

1,25

0,005

0,198

0,025

296,6

0,012

6,659

2E+08

0,075

0,085

0,004

Mean Ct value

35,63

34,1

32,74

32,61

34,98

34,72

34,29

4,815

34,26

35,47

37,86

34,47

37,06

25,25

36,96

32,94

6,72

35,7

35,53

39,41

Inf 1

Inf 2

Inf 3

Inf 4

Inf 5

Inf 6

Inf 7

Inf 8

Inf 9

Inf 10

Inf 11

Inf 12

Inf 13

Inf 14

Inf 15

Inf 16

Inf 17

Inf 18

Inf 19

Inf 20

**Cricket infection with lizard isolate at 30 ºC, study II**

**:**

mean copy numbers

mean Ct values

| **qPCR** | | **—** | **—** | **—** | **+** | **—** | **—** | **—** | **++++** | **—** | **(+)** | **—** | **—** | **—** | **++** | **—** | **+** | **++++** | **—** | **—** | **—** |
| --- | --- | --- | --- | --- | --- | --- | --- | --- | --- | --- | --- | --- | --- | --- | --- | --- | --- | --- | --- | --- | --- |
| **nPCR** | | **—** | **—** | **—** | **+** | **—** | **—** | **—** | **+** | **—** | **+** | **—** | **—** | **—** | **+** | **—** | **+** | **+** | **—** | **—** | **—** |
| **Isolation** | | **—** | **—** | **—** | **—** | **—** | **—** | **—** | **+** | **—** | **—** | **—** | **—** | **—** | **+** | **—** | **—** | **+** | **—** | **—** | **—** |
| **Iridescence** | | **—** | **—** | **—** | **—** | **—** | **—** | **—** | **+** | **—** | **—** | **—** | **—** | **—** | **—** | **—** | **—** | **+** | **—** | **—** | **—** |
| **Survival** (dpi) | | **☺** | **☺** | **☺** | **47** | **46** | **☺** | **☺** | **33** | **☺** | **56** | **☺** | **☺** | **☺** | **59** | **☺** | **54** | **40** | **☺** | **☺** | **☺** |
| **Sex** | | **♂** | **♂** | **♂** | **♂** | **♂** | **♂** | **♂** | **♂** | **♂** | **♂** | **♂** | **♂** | **♂** | **♂** | **♂** | **♂** | **♂** | **♂** | **♂** | **♂** |
| **age** | **at begin** | **ny** | **ny** | **ny** | **ny** | **ny** | **ny** | **ny** | **ny** | **ny** | **ny** | **ny** | **ny** | **ny** | **ny** | **ny** | **ny** | **ny** | **ny** | **ny** | **ny** |
|  | **at end** | **ad** | **ad** | **ad** | **ad** | **ad** | **ad** | **ad** | **ad** | **ad** | **ad** | **ad** | **ad** | **ad** | **ad** | **ad** | **ad** | **ad** | **ad** | **ad** | **ad** |

0,1

100

100000

100000000

1E+11

0

5

10

15

20

25

30

35

40

Mean copy No

15,37

4E+07

5,109

7,824

4,676

31,41

1E+09

1,403

0,473

0,401

0,234

60,7

13,67

6E+08

1E+09

4244

9E+08

1E+09

2E+09

35,77

Mean Ct value

29,9

12,49

31,2

30,69

31,3

29,06

8,301

32,71

33,99

34,19

34,82

28,29

30,04

9,321

8,352

23,3

8,929

8,424

8,138

28,91

Inf 1

Inf 2

Inf 3

Inf 4

Inf 5

Inf 6

Inf 7

Inf 8

Inf 9

Inf 10

Inf 11

Inf 12

Inf 13

Inf 14

Inf 15

Inf 16

Inf 17

Inf 18

Inf 19

Inf 20

**Cricket infection with scorpion isolate 20 ºC, study I**

**:**

mean copy numbers

mean Ct values

| **qPCR** | | **+** | **++++** | **+** | **+** | **(+)** | **+** | **++++** | **(+)** | **—** | **—** | **—** | **+** | **+** | **++++** | **++++** | **++** | **++++** | **++++** | **++++** | **+** |
| --- | --- | --- | --- | --- | --- | --- | --- | --- | --- | --- | --- | --- | --- | --- | --- | --- | --- | --- | --- | --- | --- |
| **nPCR** | | **+** | **+** | **+** | **(+)** | **+** | **+** | **+** | **—** | **—** | **—** | **—** | **+** | **+** | **+** | **+** | **+** | **+** | **+** | **+** | **+** |
| **Isolation** | | **—** | **+** | **—** | **—** | **—** | **—** | **+** | **—** | **—** | **—** | **—** | **—** | **—** | **+** | **+** | **+** | **+** | **+** | **+** | **—** |
| **Iridescence** | | **—** | **+** | **—** | **—** | **—** | **—** | **+** | **—** | **—** | **—** | **—** | **—** | **—** | **—** | **—** | **—** | **+** | **+** | **+** | **—** |
| **Survival** (dpi) | | **☺** | **52** | **☺** | **☺** | **☺** | **☺** | **31** | **46** | **☺** | **☺** | **33** | **☺** | **☺** | **31** | **37** | **☺** | **29** | **27** | **31** | **☺*** |
| **Sex** | | **♂** | **♂** | **♂** | **♂** | **♂** | **♂** | **♂** | **♂** | **♂** | **♂** | **♂** | **♂** | **♂** | **♂** | **♂** | **♂** | **♂** | **♂** | **♂** | **♂** |
| **age** | **at begin** | **ad** | **ad** | **ny** | **ny** | **ny** | **ny** | **ad** | **ad** | **ny** | **ny** | **ad** | **ad** | **ny** | **ad** | **ad** | **ny** | **ad** | **ad** | **ad** | **ny** |
|  | **at end** | **ad** | **ad** | **ad** | **ad** | **ad** | **ad** | **ad** | **ad** | **ad** | **ad** | **ad** | **ad** | **ad** | **ad** | **ad** | **ad** | **ad** | **ad** | **ad** | **ad*** |

0,1

100

100000

100000000

1E+11

0

5

10

15

20

25

30

35

40

Mean copy No

36,07

0,292

0,174

0,145

1E+09

1E+09

14,52

2E+08

12,17

0,141

10,58

2E+08

28,12

1E+09

2E+09

5,269

1,364

8,281

0,131

38,57

Mean Ct value

28,9

34,56

35,17

36,15

8,821

8,814

29,97

10,55

30,18

35,41

30,34

10,88

29,19

8,795

7,976

31,16

32,75

30,63

35,5

28,82

Inf 1

Inf 2

Inf 3

Inf 4

Inf 5

Inf 6

Inf 7

Inf 8

Inf 9

Inf 10

Inf 11

Inf 12

Inf 13

Inf 14

Inf 15

Inf 16

Inf 17

Inf 18

Inf 19

Inf 20

**Cricket infection with scorpion isolate 20 ºC, study II**

**:**

mean copy numbers

mean Ct values

| **qPCR** | | **+** | **—** | **—** | **—** | **++++** | **++++** | **+** | **++++** | **+** | **—** | **+** | **++++** | **+** | **++++** | **++++** | **+** | **(+)** | **+** | **—** | **+** |
| --- | --- | --- | --- | --- | --- | --- | --- | --- | --- | --- | --- | --- | --- | --- | --- | --- | --- | --- | --- | --- | --- |
| **nPCR** | | **+** | **—** | **—** | **—** | **+** | **+** | **+** | **+** | **+** | **—** | **+** | **+** | **+** | **+** | **+** | **+** | **—** | **(+)** | **—** | **+** |
| **Isolation** | | **—** | **—** | **—** | **—** | **+** | **+** | **—** | **+** | **—** | **—** | **—** | **+** | **—** | **+** | **+** | **—** | **—** | **—** | **—** | **—** |
| **Iridescence** | | **—** | **—** | **—** | **—** | **—** | **+** | **—** | **+** | **—** | **—** | **—** | **—** | **—** | **—** | **+** | **—** | **—** | **—** | **—** | **—** |
| **Survival** (dpi) | | **☺** | **☺** | **☺** | **☺** | **22** | **41** | **☺** | **29** | **☺** | **☺** | **☺** | **18** | **18*** | **17** | **18** | **16** | **☺** | **☺** | **☺** | **☺** |
| **Sex** | | **♂** | **♂** | **♂** | **♂** | **♂** | **♂** | **♂** | **♂** | **♂** | **♂** | **♂** | **♂** | **♂** | **♂** | **♂** | **♂** | **♂** | **♂** | **♂** | **♂** |
| **age** | **at begin** | **ny** | **ny** | **ny** | **ny** | **ny** | **ny** | **ny** | **ny** | **ny** | **ny** | **ny** | **ny** | **ny** | **ny** | **ny** | **ny** | **ny** | **ny** | **ny** | **ny** |
|  | **at end** | **ad** | **ad** | **ad** | **ad** | **ecd** | **ad*** | **ad** | **ad** | **ad** | **ad** | **ad** | **ecd*** | **ecd** | **ecd** | **ad*** | **ecd** | **ad** | **ad** | **ad** | **ad** |

Ct Mean

9,268

30,12

7,157

29,9

31,22

33,12

31,66

11,95

35,77

33,1

35,45

36,19

11,48

33,89

8,975

31,62

31,48

34,52

35,6

36,51

0,1

100

100000

100000000

1E+11

0

5

10

15

20

25

30

35

40

Quantity Mean

3E+08

57,62

1E+09

67,64

25,5

6,209

18,31

4E+07

0,867

6,306

1,094

0,634

6E+07

3,512

4E+08

18,86

20,98

2,197

0,981

0,498

Inf 1

Inf 2

Inf 3

Inf 4

Inf 5

Inf 6

Inf 7

Inf 8

Inf 9

Inf 10

Inf 11

Inf 12

Inf 13

Inf 14

Inf 15

Inf 16

Inf 17

Inf 18

Inf 19

Inf 20

**Cricket infection with scorpion isolate at 30 ºC, study I**

**:**

mean copy numbers

mean Ct values

| **qPCR** | | **++++** | **+** | **++++** | **+** | **+** | **+** | **+** | **++++** | **—** | **+** | **(+)** | **—** | **++++** | **(+)** | **++++** | **+** | **+** | **(+)** | **—** | **—** |
| --- | --- | --- | --- | --- | --- | --- | --- | --- | --- | --- | --- | --- | --- | --- | --- | --- | --- | --- | --- | --- | --- |
| **nPCR** | | **+** | **+** | **+** | **+** | **+** | **(+)** | **(+)** | **+** | **—** | **+** | **(+)** | **(+)** | **+** | **—** | **+** | **(+)** | **+** | **+** | **—** | **—** |
| **Isolation** | | **+** | **—** | **+** | **(+)** | **—** | **—** | **—** | **+** | **—** | **—** | **—** | **—** | **+** | **—** | **+** | **—** | **—** | **—** | **—** | **—** |
| **Iridescence** | | **—** | **—** | **+** | **—** | **—** | **—** | **—** | **—** | **—** | **—** | **—** | **—** | **—** | **—** | **+** | **—** | **—** | **—** | **—** | **—** |
| **Survival** (dpi) | | **11** | **53** | **19** | **52** | **☺** | **57** | **50** | **52** | **☺** | **☺** | **☺** | **☺** | **21** | **57** | **19** | **☺** | **☺** | **☺** | **46** | **53** |
| **Sex** | | **♂** | **♀** | **♀** | **♀** | **♂** | **♀** | **♀** | **♀** | **♂** | **♂** | **♂** | **♂** | **♀** | **♀** | **♂** | **♂** | **♂** | **♂** | **♀** | **♀** |
| **age** | **at begin** | **la** | **la** | **la** | **la** | **la** | **la** | **la** | **la** | **la** | **ny** | **ny** | **ny** | **la** | **la** | **la** | **ny** | **ny** | **ny** | **ny** | **ny** |
|  | **at end** | **ny** | **ad** | **ny** | **ad** | **ad** | **ad** | **ad** | **ad** | **ad** | **ad** | **ad** | **ad** | **ad** | **ad** | **ny** | **ad** | **ad** | **ad** | **ad** | **ad** |

1

1000

1000000

1000000000

1E+12

0

5

10

15

20

25

30

35

40

Quantity Mean

9E+08

158,9

46,79

18,4

71,5

61,18

2,019

72,23

1,221

20,65

1E+10

5E+07

1,378

1,751

7E+07

31,71

8,464

6E+08

1E+08

75,63

Ct Mean

7,885

28,75

30,4

31,66

29,83

30,04

34,63

29,82

31,8

28,67

6,468

11,69

35,25

32,69

8,443

28,2

32,7

9,595

10,25

27,24

Inf 1

Inf 2

Inf 3

Inf 4

Inf 5

Inf 6

Inf 7

Inf 8

Inf 9

Inf 10

Inf 11

Inf 12

Inf 13

Inf 14

Inf 15

Inf 16

Inf 17

Inf 18

Inf 19

Inf 20

**Cricket infection with scorpion isolate at 30 ºC, study II**

**:**

mean copy numbers

mean Ct values

| **qPCR** | | **++++** | **++** | **+** | **+** | **+** | **+** | **(+)** | **+** | **(+)** | **+** | **++++** | **++++** | **(+)** | **(+)** | **++++** | **+** | **+** | **++++** | **++++** | **+** |
| --- | --- | --- | --- | --- | --- | --- | --- | --- | --- | --- | --- | --- | --- | --- | --- | --- | --- | --- | --- | --- | --- |
| **nPCR** | | **+** | **+** | **+** | **+** | **+** | **+** | **(+)** | **+** | **—** | **+** | **+** | **+** | **—** | **—** | **+** | **+** | **(+)** | **+** | **+** | **+** |
| **Isolation** | | **+** | **+** | **—** | **—** | **—** | **—** | **—** | **—** | **—** | **—** | **+** | **+** | **—** | **—** | **+** | **—** | **—** | **+** | **+** | **—** |
| **Iridescence** | | **—** | **—** | **—** | **—** | **—** | **—** | **—** | **—** | **—** | **—** | **+** | **+** | **—** | **—** | **—** | **—** | **—** | **+** | **+** | **—** |
| **Survival** (dpi) | | **21** | **☺*** | **☺** | **☺** | **☺** | **30** | **30** | **☺** | **☺** | **☺** | **19** | **19** | **21** | **☺** | **59*** | **56** | **48** | **41** | **46** | **53** |
| **Sex** | | **♀** | **♂** | **♂** | **♂** | **♂** | **♂** | **♂** | **♂** | **♂** | **♂** | **♂** | **♂** | **♂** | **♂** | **♂** | **♂** | **♂** | **♂** | **♂** | **♂** |
| **age** | **at begin** | **ny** | **ny** | **ny** | **ny** | **ny** | **ny** | **ny** | **ny** | **ny** | **ny** | **ny** | **ny** | **ny** | **ny** | **ny*** | **ad** | **ad** | **ad** | **ny** | **ny** |
|  | **at end** | **ad** | **ad** | **ad** | **ad** | **ad** | **ad** | **ad** | **ad** | **ad** | **ad** | **ad** | **ad** | **ad** | **ad** | **ny*** | **ad** | **ad** | **ad** | **ad** | **ad** |

0,01

10

10000

10000000

10000000000

0

5

10

15

20

25

30

35

40

45

Quantity Mean

26,792

1,8259

2,4679

0,098

2,2167

1E+09

8,8788

0,7068

0,4504

1,9416

3,3024

1E+09

46,568

147,11

0,0662

1,1906

1,6735

25,468

2,1428

12,457

Ct Mean

29,266

32,597

32,223

36,224

34,603

7,5436

32,505

36,332

37,013

34,803

34

7,2507

28,58

27,153

39,913

35,543

35,028

29,328

34,654

31,992

Inf 1

Inf 2

Inf 3

Inf 4

Inf 5

Inf 6

Inf 7

Inf 8

Inf 9

Inf 10

Inf 11

Inf 12

Inf 13

Inf 14

Inf 15

Inf 16

Inf 17

Inf 18

Inf 19

Inf 20

**Cricket infection with cricket isolate 20 ºC, study I**

**:**

mean copy numbers

mean Ct values

| **qPCR** | | **+** | **(+)** | **(+)** | **—** | **(+)** | **++++** | **+** | **—** | **—** | **(+)** | **(+)** | **++++** | **+** | **++** | **—** | **(+)** | **(+)** | **+** | **(+)** | **+** |
| --- | --- | --- | --- | --- | --- | --- | --- | --- | --- | --- | --- | --- | --- | --- | --- | --- | --- | --- | --- | --- | --- |
| **nPCR** | | **+** | **—** | **+** | **—** | **—** | **+** | **+** | **—** | **—** | **—** | **(+)** | **+** | **+** | **+** | **—** | **—** | **(+)** | **+** | **+** | **+** |
| **Isolation** | | **—** | **—** | **—** | **—** | **—** | **+** | **—** | **—** | **—** | **—** | **—** | **+** | **—** | **(+)** | **—** | **—** | **—** | **—** | **—** | **—** |
| **Iridescence** | | **—** | **—** | **—** | **—** | **—** | **+** | **—** | **—** | **—** | **—** | **—** | **+** | **—** | **—** | **—** | **—** | **—** | **—** | **—** | **—** |
| **Survival** (dpi) | | **☺** | **☺** | **☺** | **☺** | **☺** | **21** | **☺** | **☺** | **☺** | **☺** | **☺** | **49** | **☺** | **☺** | **☺** | **☺** | **☺** | **25** | **☺** | **☺** |
| **Sex** | | **♂** | **♂** | **♂** | **♂** | **♂** | **♂** | **♂** | **♂** | **♂** | **♂** | **♂** | **♂** | **♂** | **♂** | **♂** | **♂** | **♂** | **♂** | **♂** | **♂** |
| age | **at begin** | **ny** | **ny** | **ny** | **ad** | **ny** | **ny** | **ny** | **ny** | **ny** | **ny** | **ny** | **ny** | **ny** | **ny** | **ny** | **ny** | **ny** | **ny** | **ny** | **ny** |
|  | **at end** | **ad** | **ad** | **ad** | **ad** | **ad** | **ecd** | **ad** | **ad** | **ad** | **ad** | **ad** | **ad** | **ad** | **ad** | **ad** | **ad** | **ad** | **ad** | **ad** | **ad** |

0,01

10

10000

10000000

10000000000

0

5

10

15

20

25

30

35

40

Quantity Mean

2E+09

1,1869

19,224

2E+09

3E+09

2,6874

0,0426

1E+09

1E+09

1,1706

1,2303

369,36

0,046

0,0116

0,0446

0,1954

0,548

0,5244

2E+09

19,104

Ct Mean

6,9062

31,836

28,752

6,5862

6,2598

34,312

35,52

7,2676

7,2472

35,569

31,796

26,011

35,435

36,958

35,469

33,833

35,688

32,74

7,0066

31,346

Inf 1

Inf 2

Inf 3

Inf 4

Inf 5

Inf 6

Inf 7

Inf 8

Inf 9

Inf 10

Inf 11

Inf 12

Inf 13

Inf 14

Inf 15

Inf 16

Inf 17

Inf 18

Inf 19

Inf 20

**Cricket infection with cricket isolate 20 ºC, study II**

**:**

mean copy numbers

mean Ct values

| **qPCR** | | **++++** | **(+)** | **+** | **++++** | **++++** | **(+)** | **—** | **++++** | **++++** | **(+)** | **(+)** | **++** | **—** | **—** | **—** | **—** | **—** | **—** | **++++** | **+** |
| --- | --- | --- | --- | --- | --- | --- | --- | --- | --- | --- | --- | --- | --- | --- | --- | --- | --- | --- | --- | --- | --- |
| **nPCR** | | **+** | **+** | **+** | **+** | **+** | **(+)** | **—** | **+** | **+** | **—** | **+** | **+** | **(+)** | **—** | **—** | **—** | **—** | **(+)** | **+** | **+** |
| **Isolation** | | **+** | **—** | **—** | **—** | **—** | **—** | **—** | **+** | **+** | **—** | **—** | **+** | **—** | **—** | **—** | **—** | **—** | **—** | **+** | **—** |
| **Iridescence** | | **(+)** | **—** | **—** | **—** | **—** | **—** | **—** | **+** | **+** | **—** | **—** | **—** | **—** | **—** | **—** | **—** | **—** | **—** | **+** | **—** |
| **Survival** (dpi) | | **36** | **☺** | **☺** | **37** | **37** | **49** | **58** | **39** | **38** | **☺** | **☺** | **49** | **☺** | **☺** | **☺** | **☺** | **☺** | **☺** | **39** | **53** |
| **Sex** | | **♂** | **♂** | **♂** | **♂** | **♀** | **♂** | **♂** | **♂** | **♂** | **♂** | **♂** | **♂** | **♂** | **♂** | **♂** | **♂** | **♂** | **♂** | **♂** | **♂** |
| **age** | **at begin** | **ny** | **ny** | **ny** | **ny** | **ny** | **ny** | **ny** | **ny** | **ny** | **ny** | **ny** | **ny** | **ny** | **ny** | **ny** | **ny** | **ny** | **ny** | **ny** | **ny** |
|  | **at end** | **ad*** | **ad** | **ad** | **ad** | **ad** | **ad** | **ad** | **ad** | **ad** | **ad** | **ad** | **ad** | **ad** | **ad** | **ad** | **ad** | **ad** | **ad** | **ad** | **ad** |
