# Supplementary material for: Detection and Characterization of Invertebrate Iridoviruses Found in Reptiles and Prey Insects in Europe over the Past Two Decades
Source: Viruses. 2019 Jul 2;11(7):600. doi: 10.3390/v11070600 (PMC6669658; doi:10.3390/v11070600)
Supplement: Supplementary file 1 [file viruses-11-00600-s001.zip › Suppl.Fig.1_MCP tree_final.docx]

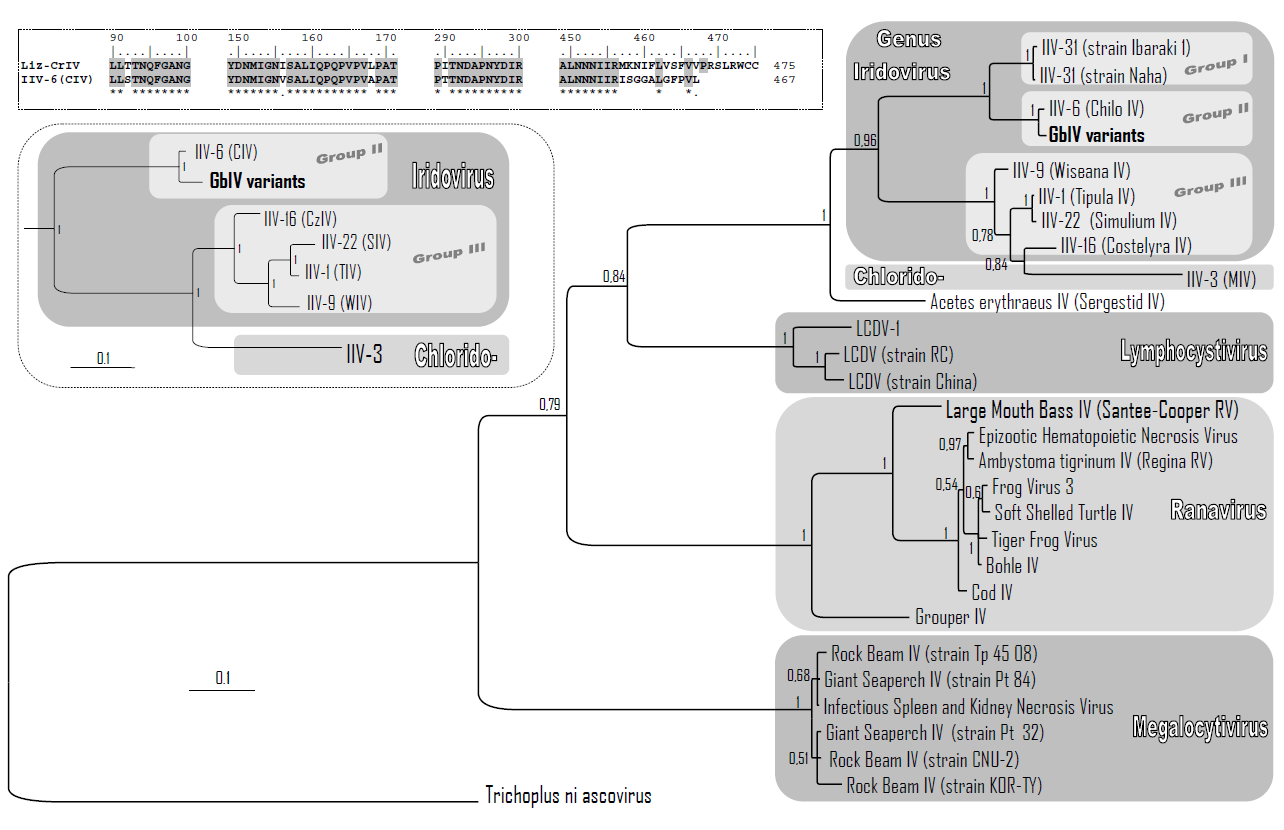


**Supplementary Figure S1.** An early phylogenetic tree reconstructions to resolve the relationship of our IIV isolates (CrIV variants; in bold face). Bayesian tree calculated from partial (347 aa) alignment of the MCPs. In the middle left inserted box, a part of the tree calculated from complete MCP sequence (less available GenBank data) to demonstrate proportional differences of CrIV/IIV6 compared to IIV1/IIV22. A partial alignment of CrIV/IIV6 capsid proteins is shown top left inserted (asterisk means identical, dot indicates conserved positions). Posterior probability values are listed on the branches. An ascovirus was used as an outgroup.

Note that at the time of these calculations (2012) the genus *Iridovirus* was an apparently paraphyletic group on the trees, and together with *Chloridovirus* member IIV3, they form a monophyletic cluster. Group III iridoviruses were later removed from the *Iridovirus* genus and assigned to the genus *Chloriridovirus*, which resolved this discrepancy.

GenBank Accesion Numbers for applied virus sequences:

ATV: AF080218,

Bohle IV: AY187046,

Cod IV: GU391284,

EHNV: AY187045,

FV3: DQ897669,

IIV-1: M39542,

IIV-3: NC_008187,

IIV-6: AF303741,

IIV-9: GQ918152,

IIV-16: AF025775,

IIV-22: M32799,

IIV-31(Ibaraki): AB686457,

IIV-31 (Naha): AB686462,

Sergestid IV: ABF82044,

LCDV-1: L63545,

LCDV (RC): AF103188,

LCDV (China): NC_005902,

Grouper IV: AY666015,

LMBIV: FR682503,

SSTIV: DQ335253,

TFV: AF389451,

RBIV (Tp4508): JF264352,

GSIV (Pt84): JF264354,

ISKNV: AB66996,

GSIV (Pt32): JF264346,

RBIV (CNU-2): AY849394,

RBIV (KOR-TY): AY532607,

Tricoplusia AV: AY197700
